# Determinants of maternal and neonatal PFAS concentrations: a review

**DOI:** 10.1186/s12940-023-00992-x

**Published:** 2023-05-10

**Authors:** Jordan McAdam, Erin M. Bell

**Affiliations:** 1 Department of Environmental Health Sciences, University at Albany, Rensselaer, NY USA; 2 Department of Epidemiology and Biostatistics, University at Albany, Rensselaer, NY USA

**Keywords:** PFOA, PFOS, PFHxS, PFNA, Determinants, Maternal health, Neonatal health

## Abstract

Per- and polyfluoroalkyl substances (PFAS) are used for their properties such as stain and water resistance. The substances have been associated with adverse health outcomes in both pregnant mothers and infants, including pre-eclampsia and low birthweight. A growing body of research suggests that PFAS are transferred from mother to fetus through the placenta, leading to in utero exposure. A systematic review was performed using the PubMed database to search for studies evaluating determinants of PFAS concentrations in blood matrices of pregnant mothers and neonates shortly after birth. Studies were included in this review if an observational study design was utilized, exposure to at least one PFAS analyte was measured, PFAS were measured in maternal or neonatal matrices, at least one determinant of PFAS concentrations was assessed, and results such as beta estimates were provided. We identified 35 studies for inclusion in the review and evaluated the PFAS and determinant relationships among the factors collected in these studies. Parity, breastfeeding history, maternal race and country of origin, and household income had the strongest and most consistent evidence to support their roles as determinants of certain PFAS concentrations in pregnant mothers. Reported study findings on smoking status, alcohol consumption, and pre-pregnancy body mass index (BMI) suggest that these factors are not important determinants of PFAS concentrations in pregnant mothers or neonates. Further study into informative factors such as consumer product use, detailed dietary information, and consumed water sources as potential determinants of maternal or neonatal PFAS concentrations is needed. Research on determinants of maternal or neonatal PFAS concentrations is critical to estimate past PFAS exposure, build improved exposure models, and further our understanding on dose–response relationships, which can influence epidemiological studies and risk assessment evaluations. Given the potential for adverse outcomes in pregnant mothers and neonates exposed to PFAS, it is important to identify and understand determinants of maternal and neonatal PFAS concentrations to better implement public health interventions in these populations.

**Supplementary Information:**

The online version contains supplementary material available at 10.1186/s12940-023-00992-x.

## Background

Per- and polyfluoroalkyl substances, commonly referred to as PFAS, are highly stable chemicals that are ubiquitous in the population due to their wide use in products such as non-stick cookware, carpeting, apparel, upholstery, personal care products and cosmetics, and firefighting foams [1–4]. There are thousands of PFAS that have been identified and some are persistent in the environment, bioaccumulate in humans and animals, and have been widely detected in humans [1, 5, 6]. The primary sources of human exposure are thought to be drinking water, food, and indoor dust and air [7]. Based on the carbon chain length, PFAS can be divided into short-chain and long-chain compounds, which may impact their bioaccumulation potential and persistency. Though emerging PFAS—such as GenX and perfluorobutanoate (PFBA)–are thought to have short elimination half-lives in humans, evidence suggests that exposure to these chemicals is associated with adverse health outcomes [8–11]. Long chain PFAS are commonly referred to as legacy PFAS due to their past–or legacy–use before manufacturers transitioned to other compounds. Though two of the most studied PFAS [perfluorooctane sulfonate (PFOS) and perfluorooctanoate (PFOA)] have been phased out of production in many developed countries, concern over health effects of exposure to PFOA, PFOS, and other PFAS continues. PFOA and PFOS may be most studied because of their longer biological elimination half-lives in humans and widespread use [12]. Other PFAS such as perfluorobutane sulfonate (PFBS), perfluorohexane sulfonate (PFHxS), and perfluorononoate (PFNA) have been less frequently studied [13].

There is evidence of numerous health effects associated with PFAS exposure in adults, including dyslipidemia [14–16], changes in liver enzymes [17, 18], elevated blood pressure and hypertension [19, 20], and kidney and testicular cancer [21]. In neonates and children, there is evidence of decreases in birthweight [22, 23], neurobehavioral problems [24–27], immunosuppression [28, 29], and increased adiposity [30–32].

PFAS have been detected in pregnant mothers [33–35] and the placenta is a plausible target of PFAS [35, 36]. PFAS accumulate in the placenta and pass the placental barrier, affecting the developing embryo [37, 38]. The transplacental transfer efficiency (TTE) of PFAS appears to vary depending upon carbon chain length and functional groups of the PFAS. Studies have observed U-shaped relationships between TTEs and carbon chain length and functional groups [37, 39–46]. PFAS fetal body burden can be estimated in cord blood samples. Many studies use maternal blood during pregnancy as a proxy measure of fetal exposure, likely due to ethical and other considerations associated with newborn dried bloodspots or invasive blood draws [47, 48]. However, paired maternal blood measures and infant measures of PFAS are not equal and the distribution characteristics of individual PFAS in pregnant mothers will affect the quantity present in neonates.

In addition to epidemiology studies and risk assessment evaluations, physiologically based pharmacokinetic (PBPK) modeling is a useful tool to assess neonatal exposure to PFAS [49–51]. The models can use a one-time serum PFAS concentrations to estimate past exposure levels. While contaminated drinking water has been considered a major source of PFAS exposure among members of affected communities, serum concentration levels may vary due to inter-individual variability in drinking water intakes, non-drinking water exposures, and PFAS pharmacokinetics, as specific PFAS may act differently than others [52]. Understanding determinants of PFAS concentrations is useful to better understand inter-individual variation and develop more effective and accurate PBPK models to estimate historical PFAS exposure. Knowledge of PFAS exposure disparities will likely rise in importance as researchers work to better understand dose–response relationships. Identification of vulnerable subgroups can influence individual healthcare and public health interventions in communities affected by PFAS contamination, as well as influence risk assessment and policy-making efforts.

Further research is needed to characterize factors affecting PFAS concentrations in pregnant mothers and newborns, two populations that may face increased risk to numerous adverse health outcomes with exposure to PFAS. Here we review current epidemiological understanding of determinants of both maternal and neonatal PFAS concentrations in blood matrices, particularly determinants of legacy PFAS—PFOA, PFOS, PFHxS, and PFNA–concentrations. We use this review to summarize knowledge gaps and future research needs.

## Methods

### Data sources and search strategies

**Fig. 1:**
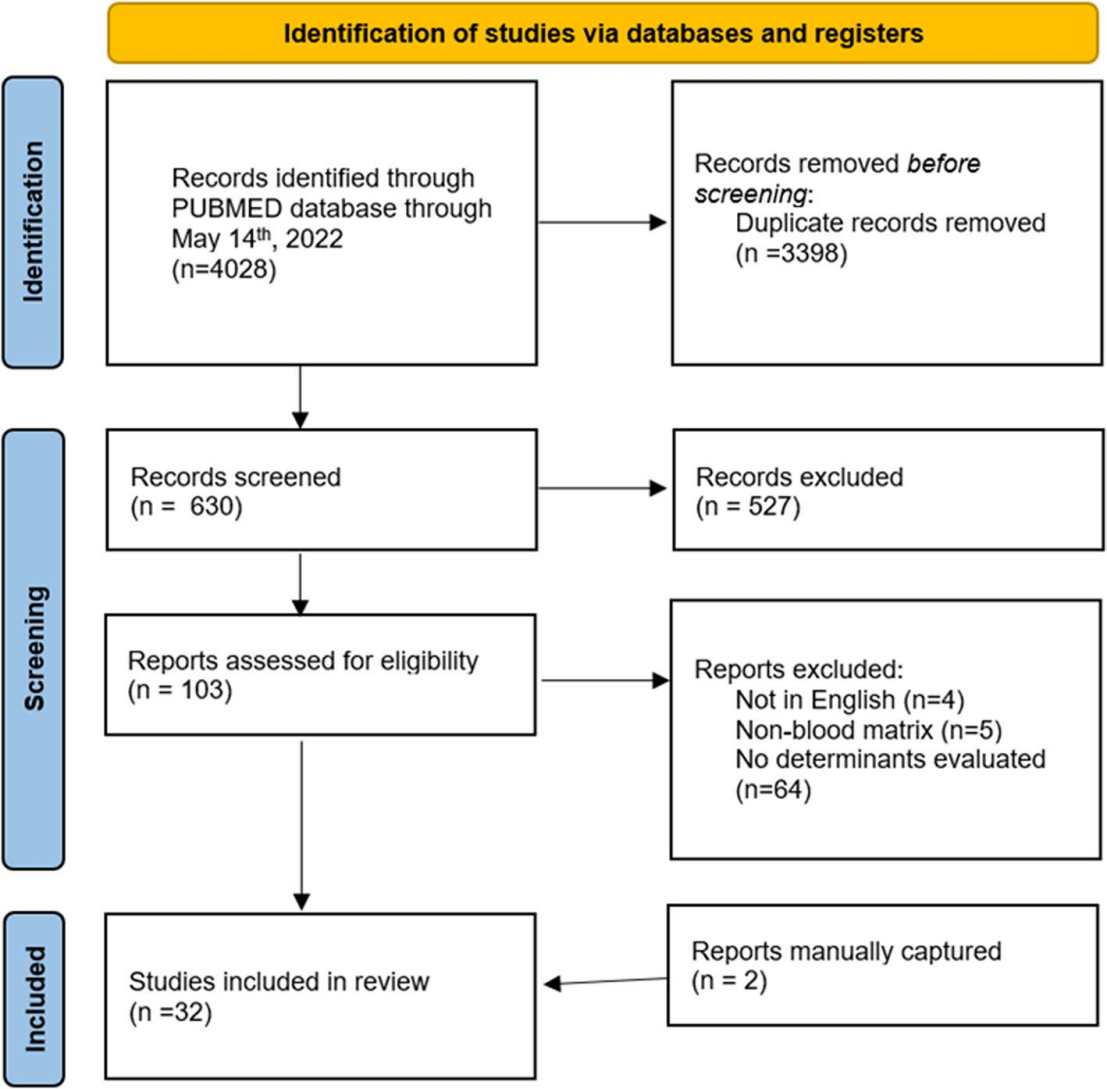
PRISMA study flow diagram and study selection criteria

This study was conducted using the Preferred Reporting Items for Systematic Reviews and Meta-Analyses (PRISMA) statement as a guide [53]. PubMed was searched for articles published within the last ten years (May 2012-December 2022). The last search was on 12 December 2022. Search terms included perfluoroalkyl, maternal, and determinants. A more detailed search strategy is presented in supplementary materials (Supplemental Table 1). To ensure studies were not missed, we also performed a manual search of the reference lists of the retrieved studies. Once citations lists were retrieved, PRISMA was used again as a guide to identify relevant studies (Fig. 1).

### Study Selection

We identified eligible epidemiological articles through an initial screening of the titles and abstracts found through searches. After the initial screening, full-text articles were evaluated. Studies were included if they met the following criteria: 1) An observational study design was utilized; 2) Exposure to at least one PFAS (e.g., perfluoroalkyl acids [PFAAs], perfluoroalkyl ether sulfonic acid [PFESAs] or perfluoroalkane sulfonamide substance was measured and; 3) Legacy or alternative PFAS exposure was measured during pregnancy using a maternal blood sample (e.g., serum, plasma, or whole blood) or PFAS exposure was measured in neonates using a blood sample (e.g. newborn dried bloodspots; umbilical cord blood, serum, or plasma); 4) at least one determinant of PFAS exposure was assessed in pregnant mothers or neonates; 5) results were provided, such as risk estimates, beta estimates, or percent change. We excluded: 1) studies that were not published in English, 2) laboratory studies, nonhuman studies, letters, or review articles, (3) articles assessing only health outcomes and confounders, not determinants related to PFAS exposure, and (4) articles measuring PFAS concentrations in only urine or other non-blood matrices.

PFOA, PFOS, PFHxS, and PFNA are among the most commonly studied PFAS [52, 54], therefore these were the focus of this review. Research regarding determinants of other PFAS is sparse. We include less-studied PFAS to summarize our current understanding of determinants of these chemicals and highlight the need for further study on this topic.

Many review papers focus solely on longitudinal studies due to their strengths compared to cross-sectional studies, such as the ability to infer causation [55]. However, most of the research related to determinants of PFAS concentrations in neonates and pregnant mothers is cross-sectional. Important research–such as the C8 Health Study and analyses from the nationally representative U.S. National Health and Nutrition Examination Survey (NHANES)–that has influenced our knowledge of PFAS exposure and health outcomes has been cross-sectional [56, 57]. For these reasons, cross-sectional studies were included in this review. A validated tool to evaluate the methodological quality of observational studies is lacking [58]; therefore, we consider the findings of longitudinal studies most reflective of the true relationship between PFAS exposure and potential determinants and assess whether cross-sectional results do or do not support those findings.

### Data extraction

For all eligible studies, the following was extracted: first author; year of publication; study location; study period; number of pregnant mothers, neonates, or dyads studied; mean age or average age statistic; PFAS measured; maternal factors ordeterminants evaluated; neonatal factors or determinants evaluated; maternal or neonatal factor collection method (e.g., questionnaire, biological sample, etc.); statistical analysis methods; beta estimates, adjusted beta estimates, odds ratios, relative ratios, hazard ratios, percent change, or other results with 95% confidence intervals (CIs) or *p* values; adjusted variables; and main conclusions (Supplemental Table 1).

## Results and discussion

### Study search

There was a total of 4,028 articles identified through PubMed published prior to 12 December 2022 and after May 2012. 3,398 records were duplicate citations. To be considered duplicates, two or more citations had to share the same author, title, publication date, volume, and issue [59]. After de-duplication, this left 630 records for screening. Thirty-five studies met the inclusion criteria (Fig. 1). The studies were conducted in 15 countries: Canada [60–63], Taiwan [64], Norway [65–71], Sweden [67, 72, 73], United States [42, 74–80], Slovakia [81], Japan [82], Spain [83], France [84], Greenland [85], China [86–90], Belgium [91], Brazil [92], the United Kingdom [71], and Denmark [93]. One study evaluated nine European cohorts with data on PFAS concentrations measured in the blood of pregnant mothers or in neonatal cord blood, which was divided into groups. The first group consisted of five cohorts with PFAS concentrations measured in pregnant mothers between 2003 and 2011 from France, Spain, Norway. The second group consisted of four cohorts with PFAS concentrations measured in cord blood between 2008 and 2014 from Belgium and Slovakia [94].

The characteristics of the eligible and included studies are described in Supplemental Table 1. The population sizes for the reviewed studies ranged from *n* = 66 neonates [90] to *n* = 5897 pregnant mothers [94]. Twenty-one studies were cross-sectional, while fourteen were longitudinal. PFAS exposure was assessed using maternal serum samples (*n* = 12), maternal plasma samples (*n* = 9), maternal serum and plasma (*n* = 1), cord plasma (*n* = 2), maternal and cord plasma (*n* = 2), maternal and cord serum (*n* = 5), and cord serum (*n* = 3). One study evaluated several European cohorts, where matrices varied by cohort. These included: maternal plasma, maternal serum, cord plasma, and cord serum [94]. Most studies provided a mean or range of gestational weeks when the maternal samples were collected. The mean gestational time or set time of sample collection was during the second trimester (*n* = 15), first trimester (*n* = 8), third trimester or at delivery (*n* = 6), or not described (*n* = 2) for maternal samples. For cord samples, studies described their collection shortly following birth (within 48 h of birth). Evaluated PFAS included perfluoroalkyl carboxylic acids (PFCAs), perfluoroalkane sulfonic acids (PFSAs), PFESAs, and N-alkyl perfluoroalkane sulfonamides (N-alkyl FASAs). The PFAS that were examined in each study, along with the sample matrices, sample collection period, maternal or infant factors or determinants, statistical methods, adjusted confounders, and findings for each study are presented in Supplemental Table 1.

Candidate determinants of maternal or neonatal PFAS levels evaluated by the studies included are summarized in Table 1. In addition, several dietary determinants were evaluated, presented in Table 2.

**Table 1:** Number of studies (*n* = 35) evaluating candidate determinants of PFAS exposure in pregnant mothers or neonates

Predictor	Number of studies examining predictor (n)
Maternal age	27
Parity	25
Maternal BMI^a^	22
Maternal education	20
Maternal smoking or serum cotinine	20
Maternal race, nationality, or country of origin	15
Household income	12
Maternal alcohol consumption	11
Breastfeeding history	8
Infant sex	7
Marital status	7
Preterm delivery or gestational age	6
Sample collection date during collection period	5
Source of drinking water	4
Urban, rural, or suburban home	4
Housing characteristics	3
Menstruation characteristics and history	3
Personal care product use	3
Weight gain during pregnancy	3
Employment status	2
Food insecurity	2
Home renovations	2
Hospital where neonate was born	2
Oral contraceptive use	2
Airborne precursors to PFAAs (monitored through air sampling)	1
Cockroaches in the home	1
Consumption of fish oils	1
Distance to industrial plant	1
Environmental contaminants exposure in the home	1
Exposure to World Trade Center (WTC) disaster	1
Fasting status at blood draw	1
Financial strain	1
Frequency of floor cleaning	1
Maternal GFR	1
Healthy eating index	1
Maternal heavy metal concentrations	1
Home redecoration	1
Insurance status	1
Maternal weight	1
Marijuana use during pregnancy	1
Method of delivery	1
Occupation	1
Packing hot food in plastic bags	1
Paternal education	1
Maternal plasma albumin	1
Previous miscarriages	1
Silver dental fillings	1
Social class	1
Time between pregnancies	1
Use of nonstick cookware	1
Use of pesticides during pregnancy	1
Year of delivery	1

^a^ *BMI* Body mass index (kg/m^2^), *GFR* Glomerular filtration rate (per 10 ml/min per 1.73 m^2^)

**Table 2:** Number of studies (*n* = 35) evaluating dietary determinants of PFAS exposure in pregnant mothers or neonates

Predictor	N
Fish or shellfish	14
Meat	7
Fruits and vegetables	9
Dairy	8
Eggs	8
Cereals and pasta	4
Animal offal	4
Bread and crackers	3
Fast food/takeout	2
Marine mammals	2
Nutrient supplementation	2
Nuts	3
Seabirds/wild birds	2
Vegetable oil	2
Wheat	2
Butter/margarine	1
Coffee	1
Fried food	1
Imported food	1
Microwave popcorn	1
Mollusks	1
Pastries	1
Pies, pasties	1
Pizza	1
Puffed foods^a^	1
Salty snacks	1
Sausage, burgers	1
Seaweed	1
Soy products	1
Sweets, chocolates	1
Tea	1
Total calories	1
Tubers	1

^a^Study evaluating this predictor does not specify this further; however, puffed foods refer to food with loose or crisps texture, which is made from grains, potatoes, beans, fruits, nuts, or seeds by puffing process in China

Few studies assessed interactive or additive effects of PFAS or determinants. Several studies (*n* = 5) included a summed PFAS variable [60, 77, 87, 90, 93]. A small number of studies (*n* = 3) included interaction terms, such as: breastfeeding and maternal age [76]; duration and recency of oral contraceptive use [69]; menstrual cycle length and previous birth [70]; and menstrual cycle length and PFNA, perfluoroheptanesulfonic acid (PFHpS), and PFOS [70]. Most studies did not describe tests for interaction.

Findings by determinant and study design are summarized in Tables 3, 4, 5, 6, 7, 8, 9, 10 and 11. Given the differences in statistical approaches by studies in this review, null findings were defined as *p* > 0.05 or confidence intervals crossing 1. Positive and negative findings were defined as statistically significant (*p* < 0.05) or confidence intervals not crossing 1. Statistical methods, years of study, and adjusted covariates for studies are described in Supplemental Table 1.

### Maternal demographics and characteristics

#### Maternal age

##### Cohort and longitudinal studies

Maternal age was evaluated as a determinant of maternal or neonatal PFAS concentrations in 11 longitudinal studies (Table 3). Six studies evaluated maternal age as a categorical variable; four evaluated maternal age as a continuous variable. Most studies using a categorical variable used five-year intervals (i.e. < 25 years, 25 to 29 years, and ≥ 30 years), though there were exceptions: a study categorized as under 29 years and 29 years and over [81] and a study that categorized maternal age as 31 years and under and greater than 31 years [88]. Among studies evaluating maternal age as a determinant, all collected maternal measurements, except for two studies that obtained neonatal measures from cord blood only [81, 88].

###### PFOA

Of the studies (*n* = 11) that evaluated the predictive relationship between maternal age and maternal or neonatal PFOA concentrations, most (*n* = 6) studies had null findings [65, 68, 76, 81, 82, 88] (Table 3). Population sizes for the studies ranged from *n* = 100 [68] to *n* = 2123 [82]. The study observing a positive relationship had a small study population (*N* = 182); the three studies observing a negative relationship had study populations of *N* = 1195, *N* = 1983, and *N* = 1616, respectively [62, 73, 78] (Supplemental Table 1).

###### PFOS

Of the studies (*n* = 11) evaluating the predictive relationship between maternal age and PFOS levels, one study observed a negative relationship with maternal plasma measurements [78] and three studies observed a positive relationship with plasma or serum maternal PFOS measurements [42, 68, 82] (Table 3). Five studies observed null results when evaluating the relationship in mothers with plasma or serum measurements [61, 62, 65, 73, 76] and two studies observed null results in neonates with serum measurements [81, 88]. Population sizes for the studies ranged from *n* = 100 [68] to *n* = 2123 [82]. The studies with both the largest and smallest population sizes observed a positive relationship.

###### PFNA

Eight studies evaluated the predictive relationship between maternal age and maternal or neonatal PFNA levels, with mostly null (*n* = 5) findings (Table 3). Two studies observed a positive relationship [65, 78] in mothers with plasma or serum PFNA measures and one study in neonates with serum measures [81], while five studies observed null findings in mothers with plasma or serum PFNA measurements [42, 68, 73, 76, 82]. Population sizes for the studies ranged from *n* = 100 [95] to *n* = 2123 [82].

###### PFHxS

Eleven studies evaluated the predictive relationship between maternal age and maternal or neonatal PFHxS levels, with mixed findings (Table 3). Three studies observed a positive relationship with serum PFHxS measures [42, 81, 88], of which two studies used neonatal measures of PFHxS concentrations as opposed to maternal measures. These studies had smaller study populations compared to those with null findings, with study sizes of *N* = 182, *N* = 322, and *N* = 110, respectively. Three studies observed a negative relationship with maternal plasma PFHxS measures [61, 62, 78]; all had large population sizes, with study sizes of *N* = 1983, *N* = 1983, and *N* = 1668, respectively. Five articles observed null findings with serum or plasma maternal PFHxS measurements [65, 68, 73, 76, 82]. Population sizes for the studies ranged from *n* = 100 [68] to *n* = 2123 [82].

###### Additional PFAS

Nine additional PFAS were measured in maternal or neonatal serum or plasma (Table 3). In four studies evaluating the relationship between maternal age as a determinant of perfluorodecanoic acid (PFDeA) concentrations, three observed null results [68, 73, 82]. Three studies evaluating the predictive relationship between perfluoroundecanoic acid (PFUnDA) observed positive results [65, 73, 82], while two studies had null findings [68, 88]. Overall, there were few studies that evaluated each additional PFAS and findings were mixed.

##### Cross-sectional studies

Maternal age was evaluated as a determinant of maternal or neonatal PFAS concentrations in 17 cross-sectional studies (Table 3). Most studies (*n* = 10) evaluated maternal age as a categorical variable, and six studies evaluated maternal age as a continuous variable. Those using a categorical presented five-year intervals (i.e. < 25 years, 25–29 years, ≥ 30 years), though there were two clear exceptions: one study that categorized as < 28 years, 28–30 years, and > 30 years [89] and another that categorized as 16–19 years, 20–39 years, and ≥ 40 years [92]. Among studies evaluating maternal age as a determinant, all collected maternal measurements, except for three studies that obtained neonatal measures from cord blood [64, 90, 91]. Two studies evaluated determinants of both neonatal and maternal PFAS concentrations [72, 94].

###### PFOA

Of the studies (*n* = 15) that evaluated the predictive relationship between maternal age and PFOA concentrations, most (*n* = 9) studies observed null findings in mothers or neonates with serum or plasma measures [66, 67, 72, 74, 77, 83, 89, 90, 93] (Table 3). Five studies observed a positive predictive relationship between maternal age and maternal plasma [87, 94] or serum or plasma neonatal [64, 86, 91, 94] PFOA concentrations. One study observed a negative relationship between maternal age and PFOA concentrations [92], though the population was small (*N* = 135) and differed geographically from other studies in this review (Brazil). Population sizes for the studies ranged from *n* = 66 [90] to *n* = 5897 [94].

###### PFOS

Fourteen studies evaluated the predictive relationship between maternal age and PFOS concentrations; most observed (*n* = 9) null or not statistically significant results in mothers or neonates with serum or plasma measures [64, 66, 67, 72, 74, 77, 91, 92, 94] (Table 3). Six studies observed a positive predictive relationship in mothers with serum or plasma measures [83, 87, 89, 93, 94] or neonate samples [86]. One study that combined nine European cohort studies observed maternal age to be a determinant of maternal PFOS concentrations, but did not observe this relationship when evaluating the factor as a determinant of neonatal PFOS concentrations [94]. Population sizes for the studies ranged from *n* = 98 [77] to *n* = 5897 [94].

###### PFNA

Of the studies (*n* = 12) that assessed the predictive relationship between maternal age and PFNA levels, most studies (*n* = 7) observed a positive relationship [66, 86, 87, 89, 91, 93, 94] among mothers or neonates with serum or plasma measures and five studies had null findings [64, 72, 74, 77, 83] among mothers or neonates with serum or plasma measures (Table 3). Though the studies with null findings ranged in population size, one study used a principal component analysis (PCA) to assess predictive relationships between potential determinants and maternal or neonatal PFNA concentrations, unlike most studies using multivariable linear regression with factors chosen a priori or stepwise linear regression [77]. Another study with null findings used U-tests and Kruskal–Wallis tests to evaluate differences between groups, rather than using a regression analysis [72]. Population sizes for the studies ranged from *n* = 98 to *n* = 5897.

###### PFHxS

Eleven studies examined the predictive relationship between maternal age and PFHxS levels, with mixed findings (Table 3). Six observed a positive predictive relationship in mothers or neonates with or plasma measures of PFHxS [83, 86, 89, 91, 93, 94] and five had null findings in mothers or neonates with serum or plasma measures of PFHxS [66, 74, 77, 87, 90]. Four of the five studies with null findings had population sizes under *N* = 500, and one study used Kruskal–Wallis tests to evaluate differences between groups, rather than a regression analysis [90]. Population sizes for the studies ranged from *n* = 66 to *n* = 5897 [94].

###### Additional PFAS

Ten additional PFAS measured in maternal or neonatal serum or plasma were evaluated for a predictive relationship by maternal age in nine studies, with mixed finding (Table 3). Of the six studies evaluating the predictive relationship between maternal age and maternal or neonatal PFDeA concentrations, most had null findings (*N* = 4) [74, 77, 90, 93]. Seven studies evaluated the predictive relationship between maternal age and maternal or neonatal PFUnDA levels, with most having null results (*N* = 4) [64, 74, 87, 90]. Overall, there were few studies that evaluated each additional PFAS and findings were mixed.

**Table 3:** Studies evaluating maternal age as a determinant of maternal or neonatal PFAS concentrations by PFAS, study design, and findings

PFAS (study design)^a^	N	Positive findings	N	Negative findings	N	Null findings
Legacy PFAS						
PFOA (C/L)	1	[42]	4	[61, 62, 73, 78]	6	[65, 68, 76, 81, 82, 88]
PFOA (C-S)	5	[64, 86, 87, 91, 94]	1	[92]	9	[66, 67, 72, 74, 77, 83, 89, 90, 93]
PFOS (C/L)	3	[42, 68, 82]	1	[78]	7	[61, 62, 65, 73, 76, 81, 88]
PFOS (C-S)	6	[83, 86, 87, 89, 93, 94]	–	–	9	[64, 66, 67, 72, 74, 77, 91, 92, 94]
PFNA (C/L)	3	[65, 78, 81]	–	–	5	[42, 68, 73, 76, 82]
PFNA (C-S)	7	[66, 86, 87, 89, 91, 93, 94]	–	–	5	[64, 72, 74, 77, 83]
PFHxS (C/L)	3	[42, 81, 88]	3	[61, 62, 78]	5	[65, 68, 73, 76, 82]
PFHxS (C-S)	6	[83, 86, 89, 91, 93, 94]	–	–	5	[66, 74, 77, 87, 90]
Other PFAS						
Et-PFOSA-AcOH (C/L)	–	–	–	–	1	[78]
Me-PFOSA-AcOH (C/L)	–	–	1	[78]	1	[42]
NMeFOSAA (C-S)	–	–	–	–	1	[74]
PFBS (C-S)	1	[86]	–	–	–	–
PFDeA (C/L)	1	[65]	–	–	3	[68, 73, 82]
PFDeA (C-S)	2	[86, 87]	–	–	4	[74, 77, 90, 93]
PFDoA (C/L)	2	[73, 88]	–	–	1	[82]
PFDoA (C-S)	1	[87]	–	–	1	[86]
PFHpA (C/L)	–	–	–	–	2	[73, 88]
PFHpA (C-S)	–	–	–	–	1	[86]
PFHpS (C/L)	–	–	–	–	1	[65]
PFHpS (C-S)	1	[93]	–	–	–	–
PFOSA (C-S)	–	–	–	–	1	[86]
PFPeA (C-S)	–	–	–	–	2	[74, 90]
PFTrDA (C/L)	1	[82]	–	–	–	–
PFTrDA (C-S)	1	[87]	–	–	–	–
PFUnDA (C/L)	3	[65, 73, 82]	–	–	2	[68, 88]
PFUnDA (C-S)	3	[86, 89, 93]	–	–	4	[64, 74, 87, 90]
6:2 Cl-PFESA (C/L)	1	[88]	–	–	–	–
ΣPFAS (C-S)	1	[87]	–	–	1	[90]

^a^ *C/L* Cohort/longitudinal studies, *C-S* Cross-sectional studies, *PFOA* Perfluorooctanoic acid; PFOS: Perfluorooctane sulfonic acid, *PFNA* Perfluorononanoic acid, *PFHxS* perfluorohexane sulfonate, *Et-PFOSA-AcOH* 2-(N-Ethyl-perfluorooctane sulfonamido) acetic acid, *Me-PFOSA-AcOH* 2-(N-Methyl-perfluorooctane sulfonamido) acetic acid, *NMeFOSAA* N-methyl perfluorooctanesulfonamidoacetic acid, *PFBS* Perfluorobutane sulfonate, *PFDeA* Perfluorodecanoic acid, *PFDoA* Perfluorododecanoic acid, *PFHpA* Perfluoroheptanoic acid; *PFHpS* Perfluoroheptanesulfonic acid; *PFOSA* Perfluorooctanesulfonamide; PFPeA: perfluorovaleric acid, *PFTrDA* Perfluorotridecanoic acid (PFTrDA), *PFUnDA* Perfluoroundecanoic acid, *6:2 Cl-PFESA* 6:2 chlorinated polyfluorinated ether sulfonate

##### Maternal age discussion

Evaluating maternal age as a predictor of PFOA, PFOS, PFNA, and PFHxS concentrations in mothers or neonates showed mostly null results among longitudinal studies. Cross-sectional studies supported these results. All longitudinal studies with positive findings had serum PFHxS measures and all studies with negative findings has plasma PFHxS measures. However, this is likely a coincidence and not indicative of larger differences between studies with serum and plasma legacy PFAS measures, as serum and plasma ratios for these PFAS are 1:1 [96]. It should be noted, however, that most of the studies in this review occurred in Europe and North America, where the mean age of childbearing is higher than other parts of the world such as Central and Southern Asia and Latin America [97]. More studies are needed among populations in Africa, South America, and other parts of Asia to assess maternal age as a predictor of PFAS concentrations.

Legacy PFAS bind to serum albumin [98] and albumin concentrations decrease with age [99]. Additionally, glomerular filtration rate (GFR), kidney function, and activity level tend to decrease with age [100–102], so a general increase in legacy PFAS concentrations at older ages could be expected [103, 104]. Additionally, contraception use, hysterectomies, and menopause may reduce menstrual blood loss as women age and therefore reduce or eliminate a route of PFAS excretion [105], which could increase PFAS concentrations with age. On the contrary, parity, months of breastfeeding, pharmaceutical use, and weight tend to increase with age [106, 107], which we may expect to decrease legacy PFAS concentrations in mothers of older ages [108, 109]. This evidence suggests a complicated relationship between legacy PFAS concentrations and age, which is supported by the findings in this review. There may be varied environmental PFAS levels across geographic areas and over time; temporal trends of PFAS may be related to the year of peak emission, country PFAS regulations and legislation, and biological elimination half-lives in humans of the individual analytes [110]. Our review did not observe trends by study time period (i.e. differing findings between earlier studies than later studies), though evidence was limited.

#### Parity

##### Cohort and longitudinal studies

Parity information was collected in most of the longitudinal studies (*n* = 11), with most collecting parity information as a categorical variable. Categories included: 0, 1, 2, and 3–4 [65]; 0, 1, and 2 + [42, 61, 62, 76, 82]; nulliparous or multiparous [75, 78, 81]; and 1 and 2 + [88]. One study collected parity information as a continuous variable [73]. Among studies evaluating parity as a predictor of PFAS concentrations, all collected maternal measurements except for two studies that obtained neonatal measures from cord blood only [81, 88] (Supplemental Table 1).

###### PFOA

Ten studies evaluated the predictive relationship between parity and PFOA concentrations and. all observed that as parity increased, serum or plasma PFOA concentrations in mothers or neonates decreased [42, 61, 62, 65, 73, 76, 78, 81, 82, 88] (Table 4).

###### PFOS

Ten studies evaluated the predictive relationship between parity and serum or plasma maternal or neonatal PFOS concentrations, with all observing a negative relationship [42, 61, 62, 65, 73, 76, 78, 81, 82, 88] (Table 4).

###### PFNA

Nine studies evaluated the predictive relationship between parity and serum or plasma maternal or neonatal PFNA concentrations [62, 65, 73, 76, 78, 81, 82, 88], with most (*n* = 8) observing a negative relationship (Table 4). The study observing a negative relationship had the smallest population size [42]. Population sizes for the studies ranged from *n* = 182 [42] to *n* = 2123 [82].

###### PFHxS

Ten studies assessed the predictive relationship between parity and serum or plasma maternal or neonatal PFHxS concentrations [61, 62, 73, 76, 78, 81, 82, 88], with mostly consistent, negative findings (*n* = 7) (Table 4). Three studies had null findings in maternal and neonatal samples [42, 65, 88], though these studies had relatively small population sizes of *N* = 391, *N* = 182, and *N* = 110, respectively. Population sizes for the studies ranged from *n* = 182 [42] to *n* = 2123 [82].

###### Additional PFAS

Seven additional PFAS measured in maternal or neonatal serum or plasma were evaluated for a predictive association with parity (Table 4). All studies (*n* = 3) evaluating parity as a predictor of maternal serum or plasma PFDeA concentrations observed a negative predictive relationship [65, 73, 82]. Three of four studies evaluating parity as a predictor of PFDoA concentrations had null findings [73, 82, 88]. Three of four studies evaluating parity as a predictor of maternal and neonatal serum PFUnDA concentrations observed a negative predictive relationship [65, 73, 88]. Overall, there were few studies that evaluated each additional PFAS and findings were mixed. Unlike most other studies in this review, one study used a PCA to assess predictive relationships [75]. The principal component (PC) that had higher concentrations of several serum PFAS–among other environmental chemicals such as phenols, phthalates, and metals–observed that the nulliparous versus multiparous beta coefficient representing PFAS and other environmental chemical concentrations was 1.08 (0.77, 1.39).

##### Cross-sectional studies

Information on parity was collected in most studies with a cross-sectional design (*n* = 13) (Table 4). Most of the studies that evaluated the parity-PFAS predictive relationship evaluated parity as a categorical variable, with categories including: primiparous vs multiparous [64, 67, 72]; nulliparous, primiparous, and multiparous [83, 89]; nulliparous vs multiparous [66, 77, 87, 94]; no child, one child, two children, and three or more children [91]; and zero children, one to two children, and three to four children [74]. Two studies measured PFAS concentrations in neonates [64, 91]. Three studies had paired samples [72, 86, 92]. One study evaluated determinants of both neonatal and maternal PFAS concentrations, though the measures were not paired [94]. Remaining studies measured PFAS concentrations in mothers only.

###### PFOA

Thirteen studies evaluated parity as a predictor of maternal or neonatal serum or plasma PFOA concentrations, with most studies (*n* = 8) having null findings in mothers or neonates with serum of plasma PFOA measures [67, 77, 83, 86, 87, 89, 91, 92] (Table 4). Five studies observed a negative relationship in mothers or neonates with serum or plasma PFOA measures [64, 66, 72, 74, 94]. Population sizes for the studies ranged from *n* = 98 [77] to *n* = 5897. In a study involving two Flemish province monitoring cycles—FLEHS II (2007–2011) and FLEHS III (2012–2015)–, parity was considered a determinant of neonatal cord plasma PFOA concentrations in FLEHS II, but not PFOA concentrations in FLEHS III [91].

###### PFOS

Thirteen studies evaluated parity as a determinant of maternal or neonatal PFOS concentrations, with most studies (*n* = 8) having null findings in mothers and neonates with serum of plasma measurements [64, 67, 74, 77, 83, 86, 89, 92] (Table 4). Four studies observed a negative relationship in mothers and neonates with maternal or neonatal serum or plasma PFOS measurements [66, 72, 91, 94] and one study observed a positive relationship in mothers with plasma PFOS measurements [87]. Population sizes for the studies ranged from *n* = 98 [77] to *n* = 5897 [94]. Like the relationship with PFOA described above, in FLEHS II parity did not predict neonatal plasma PFOS concentrations, but in FLEHS III parity was a determinant [91].

###### PFNA

Eleven studies evaluated parity as a determinant of maternal or neonatal PFNA concentrations, with mostly null findings (*n* = 7) in mothers with serum or plasma measures [72, 74, 77, 83] and neonates with serum or plasma measures [64, 86, 91] (Table 4). Two studies observed a negative relationship in mothers or neonates with serum or plasma PFNA measurements [66, 94] and two observed a positive relationship in mothers with plasma and serum measurements, respectively [87, 89]. Population sizes for the studies ranged from *n* = 98 [77] to *n* = 5897 [94].

###### PFHxS

Ten studies evaluated parity as a determinant of PFHxS concentrations, with most (*n* = 7) studies having null findings and three studies observing a negative relationship in mothers or neonates with plasma and serum measurements [66, 74, 94] (Table 4). Population sizes for the studies ranged from *n* = 98 [77] to *n* = 5897 [94].

###### Additional PFAS

Seven additional PFAS were evaluated for a predictive relationship with parity (Table 4). Three of four studies evaluating the predictive relationship between parity and maternal or neonatal PFUnDA concentrations had null findings [64, 74, 89]. Overall, there were few studies that evaluated each additional PFAS and findings were mixed. In a study using PCA, maternal serum PFNA, PFDeA, and PFOA were grouped into PC 3, while PFOS and PFHxS were grouped into PC 4. The beta estimate for PC 3 was 0.02 (-0.21, 0.24) (*p* > 0.05). The beta estimate for PC 4 was -0.20 (-0.42, 0.03) (*p* > 0.05) [77].

**Table 4:** Studies evaluating parity as a determinant of maternal or neonatal PFAS concentrations by PFAS, study design, and findings

PFAS (study design)^a^	N	Positive findings	N	Negative findings	N	Null findings
Legacy PFAS						
PFOA (C/L)	–	–	10	[42, 61, 62, 65, 73, 76, 78, 81, 82, 88]	–	–
PFOA (C-S)	–	–	5	[64, 66, 72, 74, 94]	8	[67, 77, 83, 86, 87, 89, 91, 92]
PFOS (C/L)	–	–	10	[42, 61, 62, 65, 73, 76, 78, 81, 82, 88]	–	–
PFOS (C-S)	1	[87]	4	[66, 72, 91, 94]	8	[64, 67, 74, 77, 83, 86, 89, 92]
PFNA (C/L)	–	–	8	[62, 65, 73, 76, 78, 81, 82, 88]	1	[42]
PFNA (C-S)	2	[87, 89]	2	[66, 94]	7	[64, 72, 74, 77, 83, 86, 91]
PFHxS (C/L)	–	–	7	[61, 62, 73, 76, 78, 81, 82]	3	[42, 65, 88]
PFHxS (C-S)	–	–	3	[66, 74, 94]	7	[77, 83, 86, 87, 89, 91, 94]
Other PFAS						
Et-PFOSA-AcOH (C/L)	1	[78]	1	[65]	–	–
Me-PFOSA-AcOH (C/L)	1	[78]	–	–	1	[42]
NMeFOSAA (C-S)	–	–	–	–	1	[74]
PFDeA (C/L)	–	–	3	[65, 73, 82]	–	–
PFDeA (C-S)	1	[87]	–	–	1	[74]
PFDoA (C/L)	–	–	1	[73]	3	[73, 82, 88]
PFDoA (C-S)	–	–	–	–	2	[87, 88]
PFHpA (C/L)	–	–	–	–	1	[73]
PFHpS (C/L)	–	–	1	[65]	–	–
PFPeA (C-S)	–	–	–	–	2	[74, 77]
PFTrDA (C/L)	–	–	–	–	1	[82]
PFTrDA (C-S)	–	–	–	–	1	[87]
PFUnDA (C/L)	–	–	3	[65, 73, 88]	1	[82]
PFUnDA (C-S)	1	[87]	–	–	3	[64, 74, 89]
6:2 Cl-PFESA (C/L)	–	–	1	[88]	–	–
ΣPFAS (C-S)	1	[87]	–	–	–	–

^a^C/L: cohort/longitudinal studies; C-S: cross-sectional studies; PFOA: perfluorooctanoic acid; PFOS: perfluorooctane sulfonic acid; PFNA: perfluorononanoic acid; PFHxS: perfluorohexane sulfonate; Et-PFOSA-AcOH: 2-(N-Ethyl-perfluorooctane sulfonamido) acetic acid; Me-PFOSA-AcOH: 2-(N-Methyl-perfluorooctane sulfonamido) acetic acid; NMeFOSAA: N-methyl perfluorooctanesulfonamidoacetic acid; PFDeA: perfluorodecanoic acid; PFDoA: perfluorododecanoic acid; PFHpA: perfluoroheptanoic acid; PFHpS: perfluoroheptanesulfonic acid; PFPeA: perfluoropentanoic acid; PFTrDA: perfluorotridecanoic acid; PFUnDA: perfluoroundecanoic acid; 6:2 Cl-PFESA: 6:2 chlorinated polyfluorinated ether sulfonate

##### Parity discussion

Among cohort studies, findings supported that PFOA, PFOS, PFNA, and PFHxS concentrations decreased with increasing parity. Parity appears to be a strong predictor of maternal and neonatal legacy PFAS concentrations, likely due to trans-placental transfer, blood loss during delivery, or lactational transfer to offspring [111, 112], though it is difficult to disentangle the effects of parity versus breastfeeding on maternal and neonatal PFAS concentrations. More research is needed to better elucidate the magnitude of the effect of parity on maternal and neonatal legacy PFAS concentrations. Additionally, factors such as time between pregnancies, births of singletons versus twins, and miscarriages or terminated pregnancies should be evaluated to better understand the relationship between parity and maternal or neonatal PFAS concentrations.

Cross-sectional findings were less consistent, with a greater share of studies presenting results that were not statistically significant. Given the potential for reverse causality with cross-sectional studies and consistency in longitudinal results, we do not believe these findings best represent the predictive relationship between parity and maternal or neonatal legacy PFAS concentrations.

#### Smoking

##### Cohort and longitudinal studies

Eight studies evaluated the predictive relationship between smoking and PFAS concentrations (Table 5). Smoking was evaluated as a categorical variable in most studies, with categories including: never smoker, former smoker, and current smoker [61, 62, 78]; non-smoker, secondhand smoker, and active smoker [42, 73, 82]. Some studies developed these categories based on self-report [61, 62, 73, 78], while others quantified serum cotinine and categorized mothers using ranges of cotinine [42, 82]. One study presented smoking as a continuous variable using serum cotinine concentrations [76]. All studies evaluated PFAS concentrations in maternal matrices, except one using only cord blood to quantify PFAS [81].

###### PFOA

Eight studies evaluated the predictive relationship between smoking and maternal or neonatal PFOA concentrations, with mixed findings (Table 5). Two studies observed a positive relationship between maternal smoking and maternal plasma PFOA concentrations [78, 82], one observed a negative relationship with maternal plasma [61], and five had null findings in mothers or neonates with serum or plasma PFOA measures [42, 62, 73, 76, 81]. The studies with positive findings had large population sizes, *N* = 1668 and *N* = 2123, respectively [78, 82]. Population sizes for the studies ranged from *n* = 182 [42] to *n* = 2123 [82].

###### PFOS

Eight studies evaluated the predictive relationship between smoking and PFOS concentrations, with mixed findings (Table 5). Two studies observed that maternal smoking decreased maternal serum or plasma PFOS concentrations [42, 82], while one study observed that smoking increased maternal plasma PFOS concentrations [78]. Five studies had null findings in mothers or neonates with serum or plasma PFOS measurements [61, 62, 73, 76, 81]. Population sizes for the studies ranged from *n* = 182 [42] to *n* = 2123 [82].

###### PFNA

Six studies evaluated smoking as a determinant of PFNA concentrations, with one study observing that smoking predicted higher maternal plasma PFNA concentrations [78] and five having null findings in mothers and neonates with serum or plasma measurements [42, 73, 76, 81, 82] (Table 5). Population sizes for the studies ranged from *n* = 182 [42] to *n* = 2123 [82].

###### PFHxS

Eight studies assessed the predictive relationship between smoking and PFHxS concentrations, with mixed findings (Table 5). Three studies observed a positive predictive relationship with maternal serum or plasma PFHxS levels [62, 73, 78], while five studies had null findings in mothers or neonates with serum or plasma PFHxS measurements [42, 61, 76, 81, 82]. The studies with positive findings had large population sizes, *N* = 1983, *N* = 1668, and *N* = 1616, respectively and used self-reporting to classify smoking history. Population sizes for the studies ranged from *n* = 182 [42] to *n* = 2123 [82].

###### Additional PFAS

Seven additional PFAS were evaluated for a predictive relationship with maternal smoking history (Table 5) Both studies evaluating maternal smoking history as a determinant of PFUnDA concentrations observed a negative relationship [73, 82]. Overall, there were few studies that evaluated each additional PFAS and findings were mixed.

##### Cross-sectional studies

Most cross-sectional (*n* = 11) studies assessed the predictive relationship between smoking and maternal or neonatal PFAS concentrations, with many having null findings (Table 5). Smoking status was collected as environmental tobacco smoke (ETS) exposure during pregnancy (yes vs. no) [64, 94], serum cotinine categorized by specific ranges [67, 72], and questions about smoking status and amount smoked [66, 77, 83, 86, 87, 89, 93, 94]. Categories related to smoking status included: no smoking, 1–10 cigarettes/day, and > 10 cigarettes/day [83]; smoked before pregnant no vs yes [89]; passive smoker no vs yes [87]; nonsmoker, occasional smoker, and daily smoker [66]; never smoker and current or former smoker [77]; nonsmoker, secondhand smoker, and active smoker [72, 86]; nonsmoker, until pregnancy, and during pregnancy [93, 94]. Most (*n* = 7) studies used maternal matrices to evaluate PFAS concentrations, except for one study that assessed neonatal PFAS concentrations in cord blood only [64]. Two studies had paired samples [72, 86]. One study had both maternal and neonatal measures of PFAS, though the samples were not paired [94].

###### PFOA

Nine studies tested the predictive relationship between smoking and maternal or neonatal serum or plasma PFOA concentrations, with all observing null findings [64, 66, 67, 72, 83, 86, 87, 89, 93] (Table 5). In a study evaluating both ETS exposure and maternal smoking status as determinants of maternal and neonatal PFOA concentrations, only ETS exposure was observed to be a determinant of PFOA concentrations and only in mothers [94].

###### PFOS

Ten studies evaluated the predictive relationship between smoking and maternal or neonatal PFOS concentrations, with inconsistent observations (Table 5). Four studies [66, 67, 93, 94] observed that smoking before or during pregnancy decreased maternal serum or plasma PFOS concentrations, one study observed that ETS exposure during pregnancy increased neonatal plasma PFOS concentrations [64], and five studies had null findings in mothers or neonates with serum or plasma PFOS measures [72, 83, 86, 87, 89]. A study with both maternal and neonatal PFOS measures observed that ETS exposure predicted higher PFOS concentrations in mothers, but lower PFOS concentrations in neonates [94]. Population sizes for the studies ranged from *n* = 237 [72] to *n* = 5897 [94].

###### PFNA

Ten studies examined the predictive relationship between smoking and maternal or neonatal PFNA concentrations [64, 66, 67, 72, 83, 86, 87, 89, 93, 94] (Table 5). All but two studies, with large population sizes of *N* = 5897 and *N* = 981, respectively, [87, 94] had null findings. Population sizes for the studies ranged from *n* = 237 [72] to *n* = 5897 [94].

###### PFHxS

Eight studies assessed the predictive relationship between maternal smoking and maternal or neonatal PFHxS concentrations [66, 72, 83, 86, 87, 89, 93, 94], with most (*n* = 7) findings null (Table 5). Population sizes for the studies ranged from *n* = 237 [72] to *n* = 5897 [94].

###### Additional PFAS

Nine additional PFAS measured in maternal and neonatal serum and plasma were evaluated for a predictive relationship with maternal smoking history (Table 5). Of the four studies that evaluated the predictive relationship between maternal smoking history and PFDeA concentrations, two studies observed null findings [77, 86]. Five studies evaluated the relationship between maternal smoking history and PFUnDA concentrations, with most findings null (*n* = 3) [64, 86, 89]. Overall, there were few studies that evaluated each additional PFAS and findings were mixed.

**Table 5:** Studies evaluating cigarette smoking as a determinant of maternal or neonatal PFAS concentrations by PFAS, study design, and findings

PFAS (study design) ^a^	N	Positive findings	N	Negative findings	N	Null findings
Legacy PFAS						
PFOA (C/L)	2	[78, 82]	1	[61]	5	[42, 62, 73, 76, 81]
PFOA (C-S)	–	–	–	–	9	[64, 66, 67, 72, 83, 86, 87, 89, 93]
PFOS (C/L)	1	[78]	2	[42, 82]	5	[61, 62, 73, 76, 81]
PFOS (C-S)	1	[64]	4	[66, 67, 93, 94]	5	[72, 83, 86, 87, 89]
PFNA (C/L)	1	[78]	–	–	5	[42, 73, 76, 81, 82]
PFNA (C-S)	1	[87]	1		8	[64, 66, 67, 72, 83, 86, 89, 93]
PFHxS (C/L)	3	[62, 73, 78]	–	–	5	[42, 61, 76, 81, 82]
PFHxS (C-S)	–	–	1	[93]	7	[66, 72, 83, 86, 87, 89, 94]
Other PFAS						
Et-PFOSA-AcOH (C/L)	–	–	–	–	1	[78]
Me-PFOSA-AcOH (C/L)	–	–	–	–	2	[42, 78]
PFBS (C-S)	–	–	–	–	1	[86]
PFDeA (C/L)	–	-	1	[73]	1	[82]
PFDeA (C-S)	1		1	[93]	2	[77, 86]
PFDoA (C/L)	–	–	1	[73]	1	[82]
PFDoA (C-S)	1	[87]	–	–	1	[86]
PFHpA (C/L)	–	–	–	–	1	[73]
PFHpA (C-S)	1	[86]	–	–	–	–
PFHpS (C-S)	–	–	1	[93]	–	–
PFOSA (C-S)	–	–	–	–	1	[86]
PFTrDA (C/L)	–	–	–	–	1	[82]
PFTrDA (C-S)	1	[87]	–	–	–	–
PFUnDA (C/L)	–	–	2	[73, 82]	–	–
PFUnDA (C-S)	1	[87]	1	[93]	3	[64, 86, 89]
ΣPFAS (C-S)	1	[87]	1	[93]	–	–

^a^ *C/L* Cohort/longitudinal studies, *C-S* Cross-sectional studies, *PFOA* Perfluorooctanoic acid, *PFOS* Perfluorooctane sulfonic acid, *PFNA* Perfluorononanoic acid, *PFHxS* Perfluorohexane sulfonate, *Et-PFOSA-AcOH* 2-(N-Ethyl-perfluorooctane sulfonamido) acetic acid, *Me-PFOSA-AcOH* 2-(N-Methyl-perfluorooctane sulfonamido) acetic acid, *PFBS* Perfluorobutane sulfonate, *PFDeA* Perfluorodecanoic acid, *PFDoA* Perfluorododecanoic acid, *PFHpA* Perfluoroheptanoic acid, *PFHpS* Perfluoroheptanesulfonic acid, *PFOSA* Perfluorooctanesulfonamide, *PFTrDA* Perfluorotridecanoic acid, *PFUnDA* Perfluoroundecanoic acid

##### Maternal smoking discussion

The findings on the relationship between smoking and PFAS concentrations leaned null in longitudinal studies and cross-sectional studies corroborated these findings. These findings are consistent with studies in the general population [33, 113–115]. The relationship between smoking and PFAS concentrations may be affected by differences in lifestyle patterns between smokers and non-smokers. Research has indicated that smokers may show differences in food selections, nutrient intakes, exercise habits, and BMI, though these trends could vary globally which may explain differences in findings among studies in this review [116–118]. Additionally, many studies did not evaluate smoking intensity and PFAS concentrations through either serum cotinine or questionnaires, though existing literature does not indicate that smoking intensity may be a predictor of PFAS concentrations in mothers and their offspring [66, 67, 119].

Some studies evaluated smoking history through self-reporting in questionnaires [61, 62, 64, 66, 73, 74, 77, 78, 81, 83, 86, 87, 89, 92, 93] while others used serum cotinine levels [42, 67, 72, 76, 82]. Self-reported smoking has been shown to be an inaccurate method of identifying smokers; studies suggest that up to one quarter of pregnant smokers may be missing when collecting data via self-reporting [120–123]. Studies that used serum cotinine measures had more consistent findings. Use of self-reported smoking may misclassify and bias results toward the null. Future studies should consider use of serum cotinine measures to better classify smoking’s predictive relationship with PFAS.

#### Maternal BMI

##### Cohort and longitudinal studies

Maternal BMI, commonly calculated as weight (kg)/height (m^2^), was determined using pre-pregnancy weight for the studies in this review. Nine studies evaluated the predictive relationship between maternal BMI and any PFAS (Table 6). Most studies used a categorical variable to assess maternal BMI, with categories including: lean or underweight, normal, and overweight or obese [81, 82]; underweight to normal (BMI < 25), overweight (25 ≤ BMI ≤ 30), and obese (BMI > 30) [61]; and normal (BMI < 24.9), overweight (BMI 25–29.9), and obese (BMI ≥ 30) [42, 62]. Only two studies measured PFAS concentrations in neonates only [81, 88], with other studies instead using maternal matrices.

###### PFOA

Of the eight studies evaluating the predictive relationship between maternal BMI and maternal or neonatal PFOA concentrations, all had null findings [42, 61, 62, 65, 78, 81, 82, 88] (Table 6).

###### PFOS

Eight studies assessed the predictive relationship between maternal BMI and maternal or neonatal PFOS concentrations, with mostly null findings (Table 6). One study observed an inverse relationship [42] and two studies observed a positive predictive relationship between maternal BMI and PFOS concentrations: one with maternal plasma PFOS levels [78] and one with neonatal serum PFOS levels [88]. Two studies with significant findings had small population sizes, *N *= 182 and *N* = 110, respectively [42, 88]. Five studies had null findings: three with maternal serum or plasma measures of PFOS [61, 62, 65, 82] and one with neonatal measures of PFOS [81]. Population sizes for the studies ranged from *n* = 110 [88] to *n* = 2123 [82].

###### PFNA

Five studies assessed the predictive relationship between maternal BMI and maternal or neonatal PFNA concentrations, with mostly null findings (Table 6). Two studies observed an inverse relationship [42, 82], while another study observed an inverse-U-shaped relationship, with the highest maternal serum or plasma PFNA concentrations in mothers with a pre-pregnancy BMI between 18.5–24.9 kg/m^2^ [78]. Two studies observed null findings in mothers or neonates [65, 81]. Population sizes for the studies ranged from *n* = 322 [81] to *n* = 2123 [82].

###### PFHxS

Eight studies evaluated the relationship between maternal BMI and maternal or neonatal PFHxS concentrations, with all having null findings [42, 61, 62, 65, 78, 81, 82, 88] (Table 6).

###### Additional PFAS

Eight additional PFAS measured in maternal or neonatal serum or plasma were evaluated for a predictive relationship with maternal BMI history (Table 6). Three studies evaluated maternal BMI as a determinant of maternal or neonatal PFUnDA concentrations and two had null findings [82, 88]. Overall, there were few studies that evaluated each additional PFAS and findings were mixed. One study employed a PCA, where maternal BMI (β per SD increase in BMI =  − 0.48, 95% CI: − 0.64, − 0.33) was associated with the PC with higher concentrations of maternal serum PFAS [75].

##### Cross-sectional studies

Nearly half (*n* = 5) of the twelve cross-sectional studies evaluating maternal BMI as a determinant of maternal or neonatal PFAS concentrations used a continuous measure of BMI [66, 67, 74, 77, 86]. Among studies that evaluated maternal BMI as a categorical variable, categories included: < 24, 24–28, and > 28 [89]; normal weight, underweight, overweight, and obese [83]; < 18.5, 18.5–24.9, and ≥ 25 [87, 93]; and underweight and normal vs overweight and obese [72, 94]. One study measured PFAS in cord serum. Two studies had paired samples; one study used neonatal measures to evaluate relationships [86] and one evaluated determinants of both neonatal and maternal PFAS concentrations [72]. One study had PFAS measures from both mothers and neonates, though the samples were not paired [94]. Remaining studies used maternal matrices to measure PFAS (*n* = 8).

###### PFOA

Twelve studies assessed the predictive relationship between maternal BMI and maternal or neonatal PFOA concentrations [66, 67, 72, 74, 83, 86, 87, 89, 91–94], with most (*n* = 9) resulting in null findings (Table 6). Population sizes for the studies ranged from *n* = 135 [92] to *n* = 5897 [94] and did not appear to influence findings.

###### PFOS

Twelve studies evaluated the predictive relationship between maternal BMI and maternal or neonatal PFOS concentrations [66, 67, 72, 74, 83, 86, 87, 89, 91–94], with most (*n* = 7) resulting in null findings (Table 6). Population sizes for the studies ranged from *n* = 135 [92] to *n* = 5897 [94] and did not appear to influence findings.

###### PFNA

One study observed that increasing maternal BMI predicted higher neonatal serum PFNA concentrations [86], one study observed that increasing maternal BMI predicted lower maternal PFNA concentrations [94], and eight studies had null findings in mothers or neonates [66, 74, 83, 87, 89, 91, 93] (Table 6). One study had both maternal and neonatal measures of PFNA and observed a significant relationship only in mothers [94]. Population sizes for the studies ranged from *n* = 369 [86] to *n* = 5897 [94] and did not appear to influence findings.

###### PFHxS

The nine studies evaluating the predictive relationship between maternal BMI and PFHxS concentrations had mixed findings (Table 6). Two studies observed an inverse relationship with maternal serum or plasma PFHxS measures [74, 87], two a positive relationship with maternal serum or plasma PFHxS measures [66, 89], and five studies had null findings in maternal or neonatal serum or plasma measures [83, 86, 91, 93, 94]. Population sizes for the studies ranged from *n* = 369 [86] to *n* = 5897 [94] and did not appear to influence findings.

###### Additional PFAS

Ten additional PFAS measured in maternal or neonatal serum or plasma were evaluated for a predictive relationship with maternal BMI history (Table 6). Four studies evaluated maternal BMI as a determinant of maternal or neonatal PFDeA concentrations, with half (*n* = 2) studies having null findings [74, 87]. Five studies evaluated maternal BMI as a determinant of maternal or neonatal PFUnDA concentrations, with three studies having null findings [86, 87, 89]. Overall, there were few studies that evaluated each additional PFAS and findings were mixed.

**Table 6:** Studies evaluating maternal BMI as a determinant of maternal or neonatal PFAS concentrations by PFAS, study design, and findings

PFAS (study design) ^a^	N	Positive findings	N	Negative findings	N	Null findings
Legacy PFAS						
PFOA (C/L)	–	–	–	–	8	[42, 61, 62, 65, 78, 81, 82, 88]
PFOA (C-S)	1	[89]	2	[91, 93]	9	[66, 67, 72, 74, 83, 86, 87, 92, 94]
PFOS (C/L)	2	[78, 88]	1	[42]	5	[61, 62, 65, 81, 82]
PFOS (C-S)	1	[89]	4	[67, 74, 83, 91]	7	[66, 72, 86, 87, 92–94]
PFNA (C/L)	–	–	3	[42, 78, 82]	2	[65, 81]
PFNA (C-S)	1	[86]	1	[94]	7	[66, 74, 83, 87, 89, 91, 93]
PFHxS (C/L)	–	–	–	–	8	[42, 61, 62, 65, 78, 81, 82, 88]
PFHxS (C-S)	2	[66, 89]	2	[74, 87]	5	[83, 86, 91, 93, 94]
Other PFAS						
Et-PFOSA-AcOH (C/L)	1	[78]	–	–	–	–
Me-PFOSA-AcOH (C/L)	1	[78]	–	–	1	[42]
NMeFOSAA (C-S)	–	–	–	–	1	[74]
PFDeA (C/L)	–	–	2	[65, 82]	–	–
PFDeA (C-S)	1	[86]	1	[93]	2	[74, 87]
PFDoA (C/L)	–	–	1	[82]	1	[88]
PFDoA (C-S)	1	[86]	–	–	1	[87]
PFHpA (C-S)	1	[86]	–	–	–	–
PFHpS (C/L)	–	–	–	–	1	[65]
PFHpS (C-S)	–	–	–	–	1	[93]
PFOSA (C-S)	–	–	–	–	1	[86]
PFPeA (C-S)	–	–	–	–	1	[74]
PFTrDA (C/L)	–	–	1	[82]	–	–
PFTrDA (C-S)	–	–	–	–	1	[87]
PFUnDA (C/L)	–	–	1	[65]	2	[82, 88]
PFUnDA (C-S)	–	–	2	[74, 93]	3	[86, 87, 89]
6:2 Cl-PFESA (C/L)	–	–	1	[88]	–	–
ΣPFAS (C-S)	–	–	–	–	1	[87]

^a^ *C/L* Cohort/longitudinal studies, *C-S* Cross-sectional studies, *PFOA* Perfluorooctanoic acid, *PFOS* Perfluorooctane sulfonic acid, *PFNA* Perfluorononanoic acid, *PFHxS* Perfluorohexane sulfonate, *Et-PFOSA-AcOH* 2-(N-Ethyl-perfluorooctane sulfonamido) acetic acid, *Me-PFOSA-AcOH* 2-(N-Methyl-perfluorooctane sulfonamido) acetic acid, *NMeFOSAA* N-methyl perfluorooctanesulfonamidoacetic acid, *PFDeA* Perfluorodecanoic acid, *PFDoA* Perfluorododecanoic acid, *PFHpA* Perfluoroheptanoic acid, *PFHpS* Perfluoroheptanesulfonic acid, *PFOSA* Perfluorooctanesulfonamide, *PFPeA* Perfluoropentanoic acid, *PFTrDA* Perfluorotridecanoic acid, *PFUnDA* Perfluoroundecanoic acid, *6:2 Cl-PFESA* Chlorinated polyfluorinated ether sulfonate

##### Maternal BMI discussion

Findings on the predictive relationship between maternal BMI and maternal and neonatal PFAS concentrations were mostly null in longitudinal studies. These results were corroborated by cross-sectional study findings. Similar results have been observed in the relationship between BMI as a predictor of PFAS concentrations in the general population [124–126]. Legacy PFAS, unlike lipophilic persistent organic pollutants (POPs), are distributed to protein-rich compartments in the body like the liver, kidneys, and blood, with lower amounts accumulating in adipose tissue [127]. Dilution effects may be less significant for legacy PFAS than lipophilic substances. Adipose tissue serves as way to store energy through lipids and to regulate lipids, while PFAS are activators of peroxisome proliferator-activated receptors (PPARs), necessary for lipid metabolism.

Experimental data has suggested obesogenic effects following PFAS exposure [128] and a meta-analysis of 10 prospective studies observed gestational PFOA exposure was associated with greater adiposity in childhood [129]. A proposed mechanism has been through activation of PPARα/γ, which can increase adipocyte differentiation, stimulating fat storage [130, 131]. PFAS exposure has also been associated with disrupted thyroid function and hypothyroidism, which can increase body fat [132–134]. Lastly, as endocrine disrupting chemicals, PFAS may interact with estrogen receptors or decrease estrogen concentrations, and women with low estrogen may develop more visceral fat [135].

Several studies evaluating BMI as a predictor of maternal or neonatal PFAS concentrations used self-reported measures of pre-pregnancy BMI. There is a well-documented downward bias in self-reporting of weight, which can lead to as many as one in six to seven obese individuals as nonobese due to underestimation of BMI [136]. Regardless the survey mechanism, individuals tend to overestimate their height and underestimate their weight [137–141]. This underestimation of weight is more pronounced in women and overweight individuals [142, 143]. This phenomenon may influence results, resulting in misclassification with a bias towards the null.

BMI can be influenced by food choices, but may also be influenced by socioeconomic status, social support, food insecurity and availability, health factors, and geographic factors [144, 145]. The etiologies of BMI may affect legacy PFAS concentrations, rather than BMI itself, which could explain findings in this review and mixed results among studies evaluating this relationship in the general population. The obesogenic effects of PFAS may also impact this relationship and cross-sectional studies cannot properly capture this, given that increased adiposity generally occurs over time. Future work is needed to clarify the relationship between maternal BMI and maternal and neonatal PFAS concentrations.

#### Education, household income, and socioeconomic status

Education and household income are often used as proxy measures of socioeconomic status (SES) [146, 147]. Some articles in this review used only one proxy measure, while others used multiple factors to assess SES.

##### Cohort and longitudinal studies

Maternal education was evaluated as a determinant of maternal or neonatal PFAS concentrations in six studies [42, 61, 75, 78, 81, 82]. Seven studies assessed income as a determinant of maternal PFAS concentrations [42, 61, 62, 75, 76, 78, 82]. One study measured PFAS in neonatal samples [81] and remaining studies used maternal samples to quantify PFAS. 

###### PFOA

a Maternal education
  Five studies used education as a proxy measure of socioeconomic status and assessed its predictive relationship with PFOA concentrations, with mixed findings (Table 7). One study observed an inverse relationship between maternal education and maternal plasma PFOA concentrations [78]. Four studies had null findings in mothers or neonates with serum or plasma PFOA measurements [42, 61, 81, 82]. Population sizes for the studies ranged from *n* = 182 [42] to *n* = 2123 [82] and did not appear to influence findings.
b Household income
  Six studies used income as a proxy measure of socioeconomic status and assessed the predictive relationship between income and PFOA concentrations (Table 7). All observed that increased household income predicted greater maternal serum or plasma PFOA concentrations [42, 61, 62, 76, 82], except for one study observing an inverse relationship [78]. Population sizes for the studies ranged from *n* = 182 [42] to *n* = 2123 [82] and did not appear to influence findings.

###### PFOS

a Maternal education
  Among the five studies that evaluated the predictive relationship between maternal education and maternal or neonatal PFOS concentrations, two studies observed an inverse relationship in mothers with plasma measures of PFOS [61, 78], one observed a positive relationship in mothers with plasma measures of PFOS [82], and two studies had null findings in mothers or neonates with serum measures of PFOS [42, 81] (Table 7). The three studies with significant findings had population sizes greater than *N* = 1500. Population sizes for the studies ranged from *n* = 182 [42] to *n* = 2123 [82].
b Household income
  Six studies evaluated the predictive relationship between income and maternal or neonatal serum or plasma PFOS concentrations, with all finding a positive relationship (Table 7).

###### PFNA

a Maternal education
  Four studies evaluated the predictive relationship between maternal education and PFNA concentrations, with two observing a positive relationship in mothers or neonates with serum or plasma PFNA measures [81, 82] and two studies with null findings in mothers or neonates with serum PFNA measures [42, 78] (Table 7). Population sizes for the studies ranged from *n* = 182 [42] to *n* = 2123 [82] and did not appear to influence findings.
b Household income
  Four studies observed that increasing income predicted greater maternal serum or plasma PFNA concentrations [42, 76, 78, 82] (Table 7).

###### PFHxS

a Maternal education
  Among the five studies evaluating the predictive relationship between maternal education and PFHxS concentrations, there were mixed findings (Table 7). Two studies observed that increased maternal education predicted lower maternal serum or plasma PFHxS concentrations [42, 82] and one study observed a negative relationship [78]. Two studies did not observe a significant predictive relationship in mothers or neonates with serum or plasma measures of PFHxS [61, 81]. Population sizes for the studies ranged from *n* = 182 [42] to *n* = 1983 [61] and did not appear to influence findings.
b Household income
  Like education, the six studies evaluating the predictive relationship between income and PFHxS had mixed findings (Table 7). Four studies observed a positive predictive relationship [42, 76, 78, 82] between household income and maternal serum or plasma PFHxS concentrations, and two studies did not observe a significant relationship in mothers with plasma measures of PFHxS [61, 62]. The two studies observing a negative relationship involved the same study population. Population sizes for the studies ranged from *n* = 182 [42] to *n* = 2123 [82] and did not appear to influence findings.

######  Additional PFAS

a Maternal education
  Six additional PFAS measured in maternal serum or plasma were evaluated for a predictive relationship with maternal education (Table 7). Overall, there were few studies that evaluated each additional PFAS and findings were mixed. Maternal education was evaluated in a PCA analysis, but maternal education was not associated with PC1 scores (the cluster that was characterized by higher concentrations of maternal serum PFAS) [75].
b Household income
  Six additional PFAS measured in maternal serum or plasma were evaluated for a predictive relationship with household income in three studies. Overall, there were few studies that evaluated each additional PFAS and findings were mixed. Like maternal education, household income was not associated with PC1 scores in an PCA study, where PC1 scores were characterized by higher maternal serum PFAS concentrations [75].

##### Cross-sectional studies

Education was evaluated as a determinant of maternal or neonatal PFAS concentrations by twelve studies [64, 66, 67, 74, 83, 86, 87, 89, 90, 92–94] (Table 7). Seven studies evaluated income as a determinant of PFAS concentrations [64, 66, 74, 86, 87, 89, 92]. One study evaluated social class as a measure of socioeconomic status to predict PFAS concentrations [83]. Two studies measured PFAS concentrations in neonates only [64, 90] and two studies had paired samples [86, 92].One study had PFAS measures from both mothers and neonates, though the samples were not paired [94]. All other studies had maternal PFAS measures only.

###### PFOA

a Maternal education
  Among the twelve studies evaluating the predictive relationship between maternal education and maternal or neonatal PFOA concentrations [64, 66, 67, 74, 83, 86, 87, 89, 90, 92–94], most studies (*n* = 10) had null findings (Table 7). Population sizes ranged from *n* = 66 [90] to *n* = 5897 [94] and did not appear to influence findings.
b Household income
  Seven studies assessed the predictive relationship between income and maternal or neonatal PFOA concentrations, though five had null findings in mothers or neonates with serum or plasma PFOA measures [64, 74, 86, 89, 92] (Table 7). Two studies observed a positive predictive relationship between maternal plasma PFOA concentrations and household income [66, 87]. Population sizes ranged from *n* = 135 [92] to *n* = 534 [89] and did not appear to influence findings.

###### PFOS

a Education
  Of the eleven studies evaluating the predictive relationship between maternal education and PFOS concentrations, six had findings that were not significant in mothers or neonates with serum or plasma measures of PFOS [67, 74, 83, 86, 92, 94], two observed a positive relationship in mothers with plasma measures of PFOS [66, 87], and three observed an inverse relationship in mothers or neonates with serum or plasma measures of PFOS [64, 89, 93] (Table 7). Population sizes ranged from *n* = 135 [92] to *n* = 5897 [94] and did not appear to influence findings.
b Household income
  Seven studies assessed the predictive relationship between income and maternal or neonatal serum or plasma PFOS concentrations; all had null findings [64, 66, 74, 86, 87, 89, 92] (Table 7).

###### PFNA

a Education
  Four studies observed that greater maternal education predicted higher maternal serum or plasma PFNA concentrations [66, 74, 87, 94]. Five studies had null findings in mothers or neonates [64, 83, 86, 89, 93]. Population sizes ranged from *n* = 369 [86] to *n* = 5897 [94] and did not appear to influence findings.
b Household income
  Six studies assessed the predictive relationship between income and maternal or neonatal serum or plasma PFNA concentrations [64, 66, 74, 86, 89]; all had null findings, except for one study that observed an inverse relationship between household income and maternal plasma PFNA concentrations in a bivariate analysis [87] (Table 7). Population sizes ranged from *n* = 369 [86] to *n* = 981 [87] and did not appear to influence findings.

###### PFHxS

a Education
  Most (*n* = 6) findings evaluating the predictive relationship between maternal education and maternal or neonatal PFHxS concentrations were null; however, two studies observed a positive predictive relationship in mothers with plasma PFHxS measures and neonates with serum PFHxS measures, respectively [66, 90]. Another study observed a negative predictive relationship in mothers with serum PFHxS measures [93]. Population sizes ranged from *n* = 66 [90] to *n* = 5897 [94] and did not appear to influence findings (Table 7).
b Household income
  All (*n* = 5) studies that evaluated the predictive relationship between household income and maternal or neonatal PFHxS concentrations did not have significant results [66, 74, 86, 87, 89] (Table 7).

###### Additional PFAS

a Education
  Eleven additional PFAS measured in maternal or neonatal serum or plasma were evaluated for a predictive relationship with maternal education (Table 7). Findings were mixed. In seven studies evaluating the predictive relationship between maternal education and PFUnDA concentrations, most (*n* = 4) had null findings [64, 74, 86, 89]. Five studies evaluated the relationship between maternal education and PFDeA concentrations, with two observing a positive relationship [87, 90], one observing a negative relationship [93], and two with null findings [74, 86]. Overall, there were few studies that evaluated each additional PFAS and findings were mixed.
b Household income
  Ten additional PFAS measured in maternal or neonatal serum or plasma were evaluated for a predictive relationship with household income (Table 7). The five studies evaluating the predictive relationship between household income and maternal or neonatal PFUnDA concentrations had null findings [64, 74, 86, 87, 89]. Overall, there were few studies that evaluated each additional PFAS and findings were mixed.

**Table 7:** Studies evaluating maternal education or household income as a determinant of maternal or neonatal PFAS concentrations by PFAS, study design, and findings

Education						
**PFAS (study design) ** ^a^	**N**	**Positive findings**	**N**	**Negative findings**	**N**	**Null findings**
Legacy PFAS						
PFOA (C/L)	–	–	1	[78]	4	[42, 61, 81, 82]
PFOA (C-S)	2	[87, 90]	–	–	10	[64, 66, 67, 74, 83, 86, 89, 92–94]
PFOS (C/L)	1	[82]	2	[61, 78]	2	[42, 81]
PFOS (C-S)	2	[66, 87]	3	[64, 89, 93]	6	[67, 74, 83, 86, 92, 94]
PFNA (C/L)	2	[81, 82]	–	–	2	[42, 78]
PFNA (C-S)	4	[66, 74, 87, 94]	–	–	5	[64, 83, 86, 89, 93]
PFHxS (C/L)	2	[42, 82]	1	[78]	2	[61, 81]
PFHxS (C-S)	2	[66, 90]	1	[93]	6	[74, 83, 86, 87, 89, 94]
Other PFAS						
Et-PFOSA-AcOH (C/L)	–	–	–	–	1	[78]
Me-PFOSA-AcOH (C/L)	–	–	–	–		[42, 78]
NMeFOSAA (C-S)	–	–	–	–	1	[74]
PFBS (C-S)	–	–	–	–	1	[86]
PFDeA (C/L)	1	[82]	–	–	–	–
PFDeA (C-S)	2	[87, 90]	1	[93]	2	[74, 86]
PFDoA (C/L)	1	[82]	–	–	–	–
PFDoA (C-S)	–	–	–	–	2	[86, 87]
PFHpA (C-S)	–	–	–	–	1	[86]
PFHpS (C-S)	–	–	–	–	1	[93]
PFOSA (C-S)	–	–	–	–	1	[86]
PFPeA (C-S)	–	–	–	–	2	[74, 90]
PFTrDA (C/L)	1	[82]	–	–	–	–
PFTrDA (C-S)	1	[87]	–	–	–	–
PFUnDA (C/L)	1	[82]	–	–	–	–
PFUnDA (C-S)	2	[87, 90]	1	[93]	4	[64, 74, 86, 89]
ΣPFAS (C-S)	–	–	–	–	2	[87, 90]
**Household income**						
**PFAS (study design)**	**N**	**Positive findings**	**N**	**Negative findings**	**N**	**Null findings**
Legacy PFAS						
PFOA (C/L)	5	[42, 61, 62, 76, 82]	1	[78]	–	–
PFOA (C-S)	2	[66, 87]	–	–	5	[64, 74, 86, 89, 92]
PFOS (C/L)	6	[42, 61, 62, 76, 78, 82]	–	–	–	–
PFOS (C-S)	–	–	–	–	7	[64, 66, 74, 86, 87, 89, 92]
PFNA (C/L)	4	[42, 76, 78, 82]	–	–	–	–
PFNA (C-S)	–	–	1	[87]	5	[64, 66, 74, 86, 89]
PFHxS (C/L)	4	[42, 76, 78, 82]	–	–	2	[61, 62]
PFHxS (C-S)	–	–	–	–	5	[66, 74, 86, 87, 89]
Other PFAS						
Et-PFOSA-AcOH (C/L)	–	–	–	–	1	[78]
Me-PFOSA-AcOH (C/L)	–	–	–	–	2	[42, 78]
NMeFOSAA (C-S)	–	–	–	–	1	[74]
PFBS (C-S)	–	–	–	–	1	[86]
PFDeA (C/L)	1	[82]	–	–	–	–
PFDeA (C-S)			1	[87]	2	[74, 86]
PFDoA (C/L)	–	–	–	–	1	[82]
PFDoA (C-S)	–	–	–	–	2	[86, 87]
PFHpA (C-S)	–	–	–	–	1	[86]
PFOSA (C-S)	–	–	–	–	1	[86]
PFPeA (C-S)	–	–	–	–	1	[74]
PFTrDA (C/L)	–	–	–	–	1	[82]
PFTrDA (C-S)	–	–	–	–	1	[87]
PFUnDA (C/L)	–	–	–	–	1	[82]
PFUnDA (C-S)	–	–	–	–	5	[64, 74, 86, 87, 89]
ΣPFAS (C-S)	–	–	1	[87]	–	–

^a^ *C/L* Cohort/longitudinal studies, *C-S* Cross-sectional studies, *PFOA* Perfluorooctanoic acid, *PFOS* perfluorooctane sulfonic acid, *PFNA* Perfluorononanoic acid, *PFHxS* Perfluorohexane sulfonate, *Et-PFOSA-AcOH* 2-(N-Ethyl-perfluorooctane sulfonamido) acetic acid, *Me-PFOSA-AcOH* 2-(N-Methyl-perfluorooctane sulfonamido) acetic acid, *NMeFOSAA* N-methyl perfluorooctanesulfonamidoacetic acid, *PFBS* Perfluorobutane sulfonate, *PFDeA* Perfluorodecanoic acid, *PFDoA* Perfluorododecanoic acid, *PFHpA* Perfluoroheptanoic acid, *PFHpS* Perfluoroheptanesulfonic acid, *PFOSA* Perfluorooctanesulfonamide, *PFPeA* Perfluoropentanoic acid, *PFTrDA* Perfluorotridecanoic acid, *PFUnDA* Perfluoroundecanoic acid

##### Maternal education, household income, and socioeconomic status discussion

Longitudinal findings on the predictive relationship between maternal education and maternal or neonatal PFAS concentrations leaned null, and results were corroborated by cross-sectional study findings. Household income was a consistently positive predictor of PFAS concentrations in cohort studies, but cross-sectional observations contradicted these results and were mostly null. Given the potential for reverse causality with cross-sectional studies and consistency in longitudinal results, we do not believe these findings best represent the predictive relationship between household income and maternal or neonatal PFAS concentrations.

Household income is often strongly correlated with other factors such as education and occupation, but these correlations may vary in across societies and geographic areas. Income or SES may influence the sources of PFAS that are brought into the home, such as stain-treated upholstery and carpeting and new cars. More educated adults may tend to have higher household incomes, associated with greater opportunity to use legacy PFAS-containing products such as waterproof clothing at higher prices [148], though this may not be true in all geographic areas. Findings from longitudinal studies included in this support this notion. Further study is needed to assess the role of education and household income as determinants of maternal and neonatal PFAS concentrations, and it would be beneficial for future studies to collect information on both variables. Additionally, a better definition of in-home routes of PFAS exposure would be useful in future studies.

#### Alcohol consumption

There were too few studies that evaluated maternal alcohol consumption as a predictor of maternal or neonatal PFAS concentrations to separate the studies by design. Findings were mostly consistent and null in mothers and neonates (Table 8). PFAS concentrations were quantified in cord blood in three studies [64, 81, 91]. Remaining studies used maternal measures of PFAS only.

##### PFOA

Among the studies evaluating alcohol consumption as a predictor of maternal or neonatal PFOA concentrations (*n* = 8) [64, 66, 67, 74, 81, 82, 91, 93], only two produced significant findings (Table 8). Population sizes ranged from *n* = 322 [81] to *n* = 2123 [82] and did not appear to influence findings.

##### PFOS

Eight studies assessed the predictive relationship between maternal alcohol consumption and maternal or neonatal PFOS concentrations [64, 66, 67, 74, 81, 82, 93]; seven studies had null findings in mothers and one study observed a positive relationship in neonates with plasma PFOS measures [91] (Table 8). Population sizes ranged from *n* = 322 [81] to *n* = 2123 [82] and did not appear to influence findings.

##### PFNA

Following similar trends with PFOA and PFOS, the predictive relationship between maternal alcohol consumption and maternal or neonatal PFNA concentrations was observed to be null in most studies evaluating the relationship (*n* = 5) [64, 66, 74, 81, 82] (Table 8). Two studies observed that maternal alcohol consumption predicted higher PFNA concentrations in mothers with serum PFNA measures [93] and neonates with plasma PFNA measures [91]. Population sizes ranged from *n* = 322 [81] to *n* = 2123 [82] and did not appear to influence findings.

##### PFHxS

Like trends described for PFOS, five of six studies evaluating the relationship between maternal alcohol consumption and maternal or neonatal PFHxS had null findings [66, 74, 81, 82, 93] (Table 8). Only one study observed a significant predictive relationship between maternal alcohol consumption and neonatal plasma PFHxS concentrations in an adjusted analysis [91]. Population sizes ranged from *n* = 322 [81] to *n* = 2123 [82] and did not appear to influence findings.

##### Additional PFAS

Four studies evaluated the predictive relationship between maternal alcohol consumption and seven additional PFAS measured in maternal or neonatal serum or plasma (Table 8). Four studies evaluated the predictive relationship between maternal alcohol consumption and maternal or neonatal PFUnDA concentrations, with most (*n* = 3) having null findings [64, 74, 82]. Overall, there were few studies that evaluated each additional PFAS and findings were mixed.

**Table 8:** Studies evaluating maternal alcohol consumption as a determinant of maternal or neonatal PFAS concentrations by PFAS, study design, and findings

PFAS (study design) ^a^	N	Positive findings	N	Negative findings	N	Null findings
Legacy PFAS						
PFOA	2	[91, 93]	–	–	6	[64, 66, 67, 74, 81, 82]
PFOS	1	[91]	–	–	7	[64, 66, 67, 74, 81, 82, 93]
PFNA	2	[91, 93]	–	–	5	[64, 66, 74, 81, 82]
PFHxS	1	[91]	–	–	5	[66, 74, 81, 82, 93]
Other PFAS						
NMeFOSAA	–	–	**–**	**–**	1	[74]
PFDeA	1	[93]	**–**	**–**	2	[74, 82]
PFDoA	–	–	**–**	–	1	[82]
PFHpS	1	[93]	–	–	**–**	–
PFPeA	–	–	**–**	–	1	[74]
PFTrDA	–	–	**–**	–	1	[82]
PFUnDA	1	[93]	**–**	–	3	[64, 74, 82]

^a^ *C/L* Cohort/longitudinal studies, *C-S* Cross-sectional studies, *PFOA* Perfluorooctanoic acid; PFOS: Perfluorooctane sulfonic acid, *PFNA* Perfluorononanoic acid, *PFHxS* Perfluorohexane sulfonate, *NMeFOSAA*: N-methyl perfluorooctanesulfonamidoacetic acid, *PFDeA* Perfluorodecanoic acid, *PFDoA* Perfluorododecanoic acid, *PFHpS* Perfluoroheptanesulfonic acid, *PFPeA* Perfluoropentanoic acid, *PFTrDA* Perfluorotridecanoic acid, *PFUnDA* Perfluoroundecanoic acid

##### Maternal alcohol consumption discussion

Maternal alcohol consumption, when evaluated as a predictor of PFAS concentrations, produced mostly null findings in longitudinal and cross-sectional studies. Current understanding does not suggest alcohol to be a source of PFAS exposure nor has a clear mechanism been suggested to show that alcohol may affect legacy PFAS absorption, metabolism, or distribution, which is supported by the findings of this review. Findings of a predictive relationship with alcohol may be due to lifestyle or dietary habits related to drinking, or dehydration and changes in blood circulation blood circulation influenced by alcohol consumption [149].

Alcohol consumption in the studies reviewed was collected through self-reporting. Reports indicate that mothers may underestimate their alcohol consumption during pregnancy [150], which may bias results towards the null.

#### Nationality, race, or country of origin

Several variables were used to assess the predictive relationship between race or ethnicity and maternal or neonatal PFAS concentrations. Foreign-born status [61], race or ethnicity [42, 75–78, 92], country of birth [62, 72, 83, 93], country of residence [67], native language [66], or nationality [64]. Most studies used maternal measures only of PFAS; however, one study had only cord blood measures [64] and five studies had paired measures [42, 61, 72, 76, 92].

##### Cohort and longitudinal studies

###### PFOA

In five studies evaluating the predictive relationship between maternal race or ethnicity and maternal or neonatal PFOA concentrations, three observed significant findings (*p* < 0.05) (Table 9). Two Canadian studies did not observe a significant difference in maternal plasma PFOA concentrations between Canadian-born and foreign-born mothers in adjusted and bivariate analyses, respectively [61, 62]. Three studies based in the United States observed significant predictive relationships between race and maternal serum or plasma PFOA concentrations. All three observed non-Hispanic black mothers to have the lowest PFOA concentrations and non-Hispanic white or other races to have the highest concentrations [42, 76, 78], though two papers were based on the same cohort study, the HOME Study. Population sizes ranged from *n* = 182 [42] to *n* = 1983 [61, 62] and did not appear to influence findings.

###### PFOS

All (*n* = 5) studies evaluating the predictive relationship between race and maternal or neonatal PFOS concentrations observed significant results (Table 9). Two studies observed that serum PFOS concentrations were lower in non-Hispanic black mothers compared to non-Hispanic white mothers [42, 76], though one study observed opposing results and had maternal plasma PFOS measures [78]. Based in Canada, two studies evaluating country of birth as a predictor observed that foreign-born mothers had lower plasma PFOS concentrations than Canadian-born mothers [61, 62]. Population sizes ranged from *n* = 182 [42] to *n* = 1983 [61, 62] and did not appear to influence findings.

###### PFNA

Only three studies assessed race as a predictor of maternal or neonatal PFNA concentrations, with no consistent observations [42, 76, 78] (Table 9).

###### PFHxS

Five studies evaluated nationality, race, or country of origin as a predictor of maternal or neonatal PFHxS concentrations (Table 9). Mothers born outside of Canada had lower PFHxS concentrations than Canadian-born mothers, like trends seen in PFOS above [61, 62]. One study evaluating race as a predictor did not observe significant results [78], while two other studies evaluating race observed that Black mothers had lower serum PFHxS concentrations than white mothers and mothers of other races [42, 76]. Population sizes ranged from *n* = 182 [42] to *n* = 1983 [61, 62] and did not appear to influence findings.

###### Additional PFAS

Two studies evaluated nationality, race, or country of origin as a predictor of two additional PFAS concentrations in mothers (Table 9). Overall, there were few studies that evaluated each additional PFAS and findings were mixed.

##### Cross-sectional studies

###### PFOA

Of the eight studies evaluating race as a predictor of PFOA concentrations, half (*n* = 4) had null findings in mothers or neonates [64, 66, 77, 92] (Table 9). Four studies evaluating country of birth or origin observed the term to predict PFOA concentrations in mothers [67, 72, 83, 93]. One study observed that mothers originating from Eastern Europe, Sub-Saharan Africa, and the Middle East had significantly (*p* < 0.05) lower serum PFOA concentrations than mothers from Sweden and Denmark in bivariate analyses [72]. This study had paired samples and did not observe the same trend in neonates born to mothers from these countries. Similar geographic trends were seen in mothers with serum or plasma PFOA measures in two studies [83, 93]. Population sizes ranged from *n* = 98 [77] to *n* = 1438 [93] and did not appear to influence findings.

###### PFOS

Like PFOA, half (*n* = 4) of the studies evaluating the predictive relationship between race and PFOS concentrations in mothers with serum or plasma PFOS measures had null findings [66, 77, 92, 93] (Table 9). Trends were like those described above. Population sizes ranged from *n* = 98 [77] to *n* = 1438 [93] and did not appear to influence findings.

###### PFNA

Half of studies (*n* = 3) had null findings in mothers or neonates, following trends for PFOA and PFOS [64, 66, 77] (Table 9). Population sizes ranged from *n* = 98 [77] to *n* = 1438 [93] and did not appear to influence findings.

###### PFHxS

Four of the five studies evaluating the predictive relationship between race and PFHxS concentrations observed results that were not significant in mothers or neonates [64, 66, 77, 93] (Table 9). Population sizes ranged from *n* = 98 [77] to *n* = 1438 [93] and did not appear to influence findings.

###### Additional PFAS

Two studies evaluated nationality, race, or country of origin as a predictor of three additional PFAS concentrations in mothers or neonates (Table 9). Overall, there were few studies that evaluated each additional PFAS and findings were mixed.

**Table 9:** Studies evaluating nationality, race, or country of origin as a determinant of maternal or neonatal PFAS concentrations by PFAS, study design, and findings

PFAS (study design) ^a^	N	Significant findings	N	Null findings
Legacy PFAS				
PFOA (C/L)	3	[42, 76, 78]	2	[61, 62]
PFOA (C-S)	4	[67, 72, 83, 93]	4	[64, 66, 77, 92]
PFOS (C/L)	5	[42, 61, 62, 76, 78]		
PFOS (C-S)	4	[64, 67, 72, 83]	4	[66, 77, 92, 93]
PFNA (C/L)	3	[42, 76, 78]		
PFNA (C-S)	3	[64, 83, 93]	3	[64, 66, 77]
PFHxS (C/L)	4	[42, 61, 62, 76]	1	[78]
PFHxS (C-S)	1	[83]	4	[64, 66, 77, 93]
Other PFAS				
Et-PFOSA-AcOH (C/L)	1	[78]	1	[42]
Me-PFOSA-AcOH (C/L)	1	[78]		
PFDeA (C-S)	–	–	1	[93]
PFHpS (C-S)	1	[93]	–	–
PFUnDA (C-S)	–	–	2	[64, 93]

^a^ *C/L* Cohort/longitudinal studies, *C-S* Cross-sectional studies, *PFOA* Perfluorooctanoic acid, *PFOS* Perfluorooctane sulfonic acid; *PFNA* Perfluorononanoic acid, *PFHxS* Perfluorohexane sulfonate; Et-PFOSA-AcOH: 2-(N-Ethyl-perfluorooctane sulfonamido) acetic acid, *Me-PFOSA-AcOH* 2-(N-Methyl-perfluorooctane sulfonamido) acetic acid; *PFDeA* Perfluorodecanoic acid, *PFHpS* Perfluoroheptanesulfonic acid, *PFUnDA* Perfluoroundecanoic acid

##### Nationality, race, or country of origin discussion

Cohort studies consistently observed positive predictive relationships between maternal race or country of origin, though evidence was limited. Cross-sectional studies were less consistent. Given the potential for reverse causality with cross-sectional studies, this indicates a potential predictive association between maternal race, nationality, and country of origin and PFOA, PFOS, PFNA, and PFHxS concentrations, though further research is needed to confirm these relationships. Disparities seen in legacy PFAS concentrations between races and nationality could be due from exposures that occur regionally and may be explained by cultural habits, imported foods, and lifestyle associated with the country of birth or origin [151]. Few studies in this review indicated which countries or regions foreign-born mothers were from, limiting the ability to evaluate global areas that may show significantly lower (or higher) legacy PFAS exposure. This highlights a need to better evaluate PFAS exposure globally and in diverse populations.

#### Breastfeeding history

Seven studies evaluated the predictive relationship between breastfeeding history and maternal or neonatal PFAS concentrations (Table 10). There were too few studies to evaluate studies by design. Breastfeeding history was assessed in several ways. Two studies used a categorical variable for total breastfeeding duration [nulliparous, no, 1–12 months, 13–24 months, and > 24 months; none, short term (< 4 months), long term (4–6 months), and very long term (> 6 months)] [66, 83]; four studies used a dichotomous variable (previously breastfed versus did not previously breastfeed) [42, 68, 78, 94]; one study had a continuous variable (per month) [67]. Most (*n* = 5) studies used maternal measures of PFAS only, though one study did have paired samples [42]. Another study had PFAS measures from both mothers and neonates, though the samples were not paired [94].

##### PFOA

All studies (*n* = 7) evaluating the predictive relationship between breastfeeding history and maternal or neonatal serum or plasma PFOA concentrations observed a negative relationship [42, 66–68, 78, 83, 94] (Table 10).

##### PFOS

Most studies (*n* = 6) evaluating the predictive relationship between breastfeeding history and maternal or neonatal PFOS concentrations observed a negative relationship [42, 66–68, 78, 83] (Table 10). One study did not find a significant relationship between breastfeeding history and maternal or neonatal PFOS concentrations [94]. Population sizes ranged from *n* = 100 [68] to *n* = 5897 [94] and did not appear to influence findings.

##### PFNA

Five of the six studies assessing the predictive relationship between breastfeeding history and maternal serum or plasma PFNA concentrations observed a negative relationship [66, 68, 78, 83, 94], while one study had null findings in mothers with serum PFNA measures [42]. One study that evaluated this relationship in both mothers and neonates observed a significant negative relationship in mothers, but not neonates [94]. Population sizes ranged from *n* = 100 [68] to *n* = 5897 [94] and did not appear to influence findings.

##### PFHxS

Four of the six studies investigating the predictive relationship between breastfeeding history and PFHxS concentrations observed a negative relationship in mothers or neonates with serum or plasma measures [66, 68, 78, 83], while two study had null findings in mothers or neonates with serum or plasma PFHxS measures [42, 94]. Population sizes ranged from *n* = 100 [68] to *n* = 5420 [94] and did not appear to influence findings.

##### Additional PFAS

Three studies evaluated breastfeeding history as a predictor of four additional maternal PFAS concentrations (Table 10). Overall, there were few studies that evaluated each additional PFAS and findings were mixed.

**Table 10:** Studies evaluating breastfeeding as a determinant of maternal or neonatal PFAS concentrations by PFAS, study design, and findings

PFAS (study design) ^a^	N	Positive findings	N	Negative findings	N	Null findings
Legacy PFAS						
PFOA	–	–	7	[42, 66–68, 78, 83, 94]	–	–
PFOS	–	–	6	[42, 66–68, 78, 83]	1	[94]
PFNA	–	–	5	[66, 68, 78, 83, 94]	1	[42]
PFHxS	–	–	4	[66, 68, 78, 83]	2	[42, 94]
Other PFAS						
Et-PFOSA-AcOH	–	–	–	–	1	[78]
Me-PFOSA-AcOH	–	–	–	–	2	[42, 78]
PFDeA	–	–	–	–	1	[68]
PFUnDA	–	–	–	–	1	[68]

^a^ *C/L* Cohort/longitudinal studies, *C-S* Cross-sectional studies, *PFOA* Perfluorooctanoic acid, *PFOS* Perfluorooctane sulfonic acid, *PFNA* Perfluorononanoic acid, *PFHxS* Perfluorohexane sulfonate, *Et-PFOSA-AcOH* 2-(N-Ethyl-perfluorooctane sulfonamido) acetic acid, *Me-PFOSA-AcOH*: 2-(N-Methyl-perfluorooctane sulfonamido) acetic acid, *PFDeA* Perfluorodecanoic acid, *PFUnDA* Perfluoroundecanoic acid

##### Breastfeeding discussion

Overall, breastfeeding history appears to predict lower maternal concentrations of the evaluated PFAS. Breastfeeding history as a predictor of neonatal concentrations was not assessed. Legacy PFAS have been measured in breast milk [152–154] and a relatively high transport efficiency of PFOA through lactation has been reported [44]. In one study of breast milk, a 46% decrease in maternal PFOA and 18% decrease in maternal PFOS concentrations were observed after six months of breastfeeding by analyzing repeated breast milk samples among Norwegian mothers [155]. There are few studies evaluating legacy PFAS concentrations in infants who breastfeed compared to infants who do not. In one Faroese birth cohort, duration of exclusive breastfeeding was associated with increases of most PFAS concentrations by up to thirty percent per month [156]. Another study observed that the one-month postnatal exposure to legacy PFAS via breastfeeding was much higher than prenatal exposure in utero [157]. A longitudinal study observed that duration of breastfeeding was positively associated with serum PFOA concentrations in six- to eight-year-old girls in Cincinnati and the San Francisco Bay Area [158]. Future studies should further investigate the predictive relationship between maternal breastfeeding history and neonatal PFAS concentrations and find ways to decrease PFAS exposure in reproductive age women.

The design of many environmental epidemiology studies makes it difficult to evaluate impacts of breastfeeding and breast milk consumption separate from parity, and future studies considering breastfeeding impacts on maternal and neonatal PFAS concentrations should design studies to isolate the impacts of breastfeeding. Future studies could consider repeated sample collection and attempts to confirm durations of breastfeeding. Additionally, future work should differentiate between exclusive and partial breastfeeding to address potential residual confounding.

Although these findings may cause concern among breastfeeding mothers, recent guidance from the National Academies of Science, Engineering, and Medicine (NASEM) still advises breastfeeding for most infants given the many benefits of breastfeeding for both mothers and offspring [159, 160]. In addition, PFAS may also be present in other foods, such as the water added to formula and potentially in packaged formula and baby food though this area is understudied [161]. Both the NASEM report and recent editorials have called for additional research to best understand the implications of PFAS exposure via breastmilk.

### Maternal diet

#### Fish and marine foods consumption

##### Cohort and longitudinal studies

###### PFOA

Eight studies evaluated the predictive relationship between smoking and maternal or neonatal PFOA concentrations, with mixed findings The findings on the predictive relationship between maternal fish consumption and maternal or neonatal PFOA concentrations were mostly consistent, with all studies (*n* = 6) having null findings in mothers or neonates with serum or plasma PFOA measures [60, 61, 65, 73, 76, 81] (Table 11). Population sizes ranged from *n* = 97 [60] to *n* = 1983 and did not appear to influence findings [61]. One study, using PCA, found that maternal fish consumption positively predicted the PC that was characterized by higher maternal serum concentrations of PFOA [75].

###### PFOS

Findings were mixed on the predictive relationship between maternal fish consumption and maternal or neonatal serum or plasma PFOS concentrations, with most studies (*n* = 4) having null findings [60, 61, 76, 81] and three observing that increasing maternal fish consumption predicted greater maternal serum PFOS levels [65, 73, 75] (Table 11). Population sizes ranged from *n* = 97 [60] to *n* = 1983 and did not appear to influence findings [61]. Two of the studies with positive findings were in Scandinavian populations [65, 73].

###### PFNA

The seven studies evaluating the predictive relationship between maternal fish consumption and maternal or neonatal PFNA concentrations had mixed findings (Table 11). Three studies observed that increasing maternal fish consumption predicted greater maternal serum PFNA concentrations, with two of the studies involving Scandinavian cohorts [65, 73, 75]. One study observed that as fish consumption increased, neonatal serum PFNA decreased [81]. Finally, three studies had null findings in mothers with serum or plasma PFNA measures [60, 61, 76]. Population sizes ranged from *n* = 97 [60] to *n* = 1983 [61] and did not appear to influence findings.

###### PFHxS

Observations on the predictive relationship between maternal fish consumption and maternal or neonatal PFHxS were like those seen in PFOA, with all (*n* = 6) studies having null findings in mothers or neonates with serum or plasma PFHxS measures [60, 61, 65, 73, 76, 81] (Table 11). One study, using PCA, found that maternal fish consumption positively predicted the PC that was characterized by higher maternal serum concentrations of PFHxS [75].

###### Additional PFAS

Fish consumption was evaluated as a predictor of five additional PFAS concentrations measured in maternal serum or plasma (Table 11). Overall, there were few studies that evaluated each additional PFAS and findings were mixed.

##### Cross-sectional studies

###### PFOA

Studies (*n* = 7) evaluating this relationship had mixed findings; four studies produced null results in maternal or neonatal serum or plasma [64, 83, 84, 90] (Table 11). Three studies observed that increasing fish consumption increased serum PFOS concentrations in pregnant mothers [92, 94] and neonates [86]. In a study evaluating fish consumption as a predictor of both maternal and neonatal PFOA concentrations, the relationship was significant only in maternal models [94]. Population sizes ranged from *n* = 66 [90] to *n* = 5897 [94] and did not appear to influence findings.

###### PFOS

Studies (*n* = 6) evaluating this relationship had mixed findings; two studies produced null results in mothers or neonates with serum or plasma PFOS measures [64, 92] (Table 11). Four studies observed that increasing fish consumption increased serum or plasma PFOS concentrations in pregnant mothers [83, 84, 94] and neonates [86]. In a study evaluating fish consumption as a predictor of both maternal and neonatal PFOS concentrations, the relationship was significant only in maternal models [94]. Population sizes ranged from *n* = 106 [84] to *n* = 5897 [94] and did not appear to influence findings.

###### PFNA

Studies (*n* = 5) evaluating this relationship had mixed findings; two studies produced null results in mothers [83, 84] (Table 11). Three studies [64, 86, 94] observed that consumption of fish increased serum or plasma PFNA concentrations in pregnant mothers or neonates. In a study evaluating fish consumption as a predictor of both maternal and neonatal PFOS concentrations, the relationship was significant only in maternal models [94]. Population sizes ranged from *n* = 106 [84] to *n* = 5897 [94] and did not appear to influence findings.

###### PFHxS

Most studies (*n* = 5) evaluating the relationship between maternal fish consumption and maternal or neonatal serum or plasma PFHxS concentrations produced null findings [64, 83, 84, 86, 90] (Table 11). One study observed that increasing fish consumption predicted greater PFHxS concentrations in mothers, though this relationship was not seen in neonates in the same study [94]. Population sizes ranged from *n* = 66 [90] to *n* = 5897 [94] and did not appear to influence findings.

###### Additional PFAS

Fish consumption was evaluated as a predictor of nine additional PFAS concentrations measured in maternal or neonatal serum or plasma (Table 11). Overall, there were few studies that evaluated each additional PFAS and findings were mixed.

**Table 11:** Studies evaluating fish consumption as a determinant of maternal or neonatal PFAS concentrations by PFAS, study design, and findings

PFAS (study design)^a^	N	Positive findings	N	Negative findings	N	Null findings
Legacy PFAS						
PFOA (C/L)	–	–	–	–	6	[60, 61, 65, 73, 76, 81]
PFOA (C-S)	3	[86, 92, 94]	–	–	4	[64, 83, 84, 90]
PFOS (C/L)	3	[65, 73, 75]	–	–	4	[60, 61, 76, 81]
PFOS (C-S)	4	[83, 84, 86, 94]	–	–	2	[64, 92]
PFNA (C/L)	3	[65, 73, 75]	1	[81]	3	[60, 61, 76]
PFNA (C-S)	3	[64, 86, 94]	–	–	2	[83, 84]
PFHxS (C/L)	–	–	–	–	6	[60, 61, 65, 73, 76, 81]
PFHxS (C-S)	1	[94]	–	–	5	[64, 83, 84, 86, 90]
Other PFAS						
PFBS (C-S)	–	–	–	–	1	[86]
PFDeA (C/L)	2	[65, 73]	–	–	–	–
PFDeA (C-S)	1	[86]	–	–	1	[90]
PFDoA (C/L)	1	[73]	–	–	–	–
PFDoA (C-S)	–	–	–	–	1	[86]
PFDS (C-S)	–	–	–	–	1	[90]
PFHpA (C/L)	–	–	–	–	1	[73]
PFHpA (C-S)	–	–	–	–	1	[86]
PFHpS (C/L)	–	–	–	–	1	[65]
PFOSA (C-S)	–	–	–	–	1	[86]
PFPeA (C-S)	–	–	–	–	1	[90]
PFUnDA (C/L)	2	[65, 73]	–	–	–	–
PFUnDA (C-S)	2	[86, 90]	–	-	1	[64]
ΣPFAS (C-S)	–	–	–	–	1	[90]

^a^ *C/L* Cohort/longitudinal studies, *C-S* Cross-sectional studies, *PFOA* Perfluorooctanoic acid, *PFOS* Perfluorooctane sulfonic acid, *PFNA* Perfluorononanoic acid, *PFHxS* Perfluorohexane sulfonate, *PFBS* Perfluorobutane sulfonate, *PFDeA* Perfluorodecanoic acid, *PFDoA* Perfluorododecanoic acid, *PFDS* Perfluorodecane sulfonic acid, *PFHpA* Perfluoroheptanoic acid, *PFHpS* Perfluoroheptanesulfonic acid, *PFOSA* Perfluorooctanesulfonamide, *PFPeA* Perfluoropentanoic acid, *PFUnDA* Perfluoroundecanoic acid

##### Fish and marine food consumption discussion

Fish consumption had limited evidence to support their roles as determinants of PFAS concentrations in longitudinal evidence, and similar findings were observed in cross-sectional studies. Epidemiological studies evaluating consumption of fish have shown associations with increased legacy PFAS concentrations in other populations [108, 162]. Though this review did not find consistent, significant findings to suggest fish consumption is an important determinant of PFAS concentrations in pregnant mothers and neonates, care should be taken in setting guidance for consumption of these food items for pregnant mothers due to potential for increased PFAS exposure for the fetus. There may be differences in consumption patterns across the populations of studies included in this review and there may be differences in the types of fish or shellfish consumed globally. Many studies did not specify the types of fish consumed. Fish caught in contaminated waterways may contain greater concentrations of legacy PFAS. Additionally, pregnant mothers may exercise caution and reduce or stop fish consumption during pregnancy, which may partially explain findings in this study. Past advice from the US Food and Drug Administration (FDA) focused on limiting fish consumption during pregnancy to minimize possible risks to the fetus from methylmercury [163], and those warnings may still be heeded today. In the US, it is estimated that 10% to 20% of pregnant mothers consume no fish and on average the median intake of total fish among pregnant mothers is 51.6 g per week, much less than current FDA recommendations of 224 to 336 g per week [164, 165]. Most studies in this review were not based in the United States, however, so these consumption patterns cannot be generalized to all countries represented in this review. Future studies should consider investigating differences between types of fish consumed and whether fish were locally caught or store-bought, which could explain the mixed findings observed in this review.

## General discussion and future directions

This systematic review evaluates factors that predict PFAS concentrations in pregnant mothers or neonates from peer-reviewed studies. There was strong evidence to suggest household income, maternal race, nationality, or country of origin, parity, and breastfeeding history are important determinants of legacy PFAS concentrations in pregnant mothers and neonates. There was weak, inconsistent evidence to support fish consumption, maternal age, and maternal education as determinants of maternal or neonatal legacy PFAS concentrations. There were consistently null findings observed when evaluating maternal smoking history, maternal BMI, and maternal alcohol consumption as determinants of PFOA, PFOS, PFNA, and PFHxS concentrations in mothers and neonates. Depending on the topic in study, it may not be useful to control for these three factors in epidemiological research involving PFAS exposure in pregnant mothers and neonates. This enhances our understanding of exposure and factors related to exposure among pregnant mothers and neonates, populations both associated with adverse health outcomes related to PFAS exposure. Knowledge on the most important variables to predict exposure will help researchers to choose better variables in future studies. Furthering our grasp of the toxicokinetics of PFAS—and differences between them–is critical to a better understanding of their dose–response by converting in vitro understanding of PFAS to in vivo by estimating the range of target organ doses that can be expected from external exposure scenarios [166]. This literature informs our knowledge of the overall potential for increased legacy PFAS exposure in pregnant mothers and neonates, which can help direct guidance on ways to lessen exposure among vulnerable populations.

Overall, legacy PFAS followed similar trends across most evaluated potential determinants. There were exceptions. When evaluating the predictive relationship between parity and legacy PFAS, PFHxS had less consistent findings among longitudinal studies. The sulfonate group on PFHxS has been noted as a possible interference with transplacental transfer [167], though overall there was limited evidence presented in this review to suggest differences between these PFAS. Second, the relationship between maternal BMI and PFOS and PFNA concentrations had less null findings than other legacy PFAS chemicals. Studies in the general population have found PFOS and PFHxS exposure associated with lower BMI [168] and a review found obesity and overweight to be correlated with PFAS exposure in the general populations, as well as maternal and childhood exposure [169]. Given the limited evidence presented in this review, differences in this predictive relationship among legacy PFAS warrant further investigation. Lastly, PFOS and PFNA results when evaluating the predictive relationship with maternal fish consumption were less null than other legacy PFAS. Freshwater fish typically have higher PFOS concentrations than marine systems, which may influence these findings [170].

Because cross-sectional studies might be subject to reverse causation [171], longitudinal studies are preferable when evaluating predictive relationships between environmental chemicals and maternal or neonatal factors. Exposure misclassification may occur in cross-sectional studies when a single measure of exposure is taken (i.e., serum PFAS measurements) [172], as there is variability in exposure and excretion over time that is better quantified in a longitudinal study with multiple measures of exposure. Short-chain PFAS with shorter half-lives may be especially vulnerable to exposure misclassification due to a single measure of exposure [173]. Longitudinal studies often contain more detailed information than are collected in a single questionnaire or clinic visit in a cross-sectional study. Additionally, these studies can help reduce the impact of recall error or bias, as participants usually are asked to remember events over a shorter period [174]. Well-designed longitudinal studies are necessary to better understand trends, health effects, and effectiveness of interventions to reduce PFAS exposure [52]. For these reasons, we give more focus on findings from longitudinal studies in this review.

Overall, there were differences in study population, location, time period, statistical analysis methods, and adjusted variables that may influence findings. Study populations varied in size, location, and levels of exposure and time periods of studies ranged from the later 1986–2022. Most studies used linear regression for analyses, but there were exceptions. Some studies used stepwise methods to identify variables to include in models, while others were chosen a priori. The most commonly adjusted variables included maternal age, maternal education, and parity (Supplemental Table 1). Though we did not observe specific trends between these factors and findings, care should be taken in future studies to identify methods and adjusted variables that may allow for better comparison on this topic.

Environmental chemicals such as PFAS are often studied as individual analytes, though they exist in the environment as mixtures of not only various PFAS, but with many contaminants such as pesticides, PBDEs, PCBs, and more. Studies included in this review quantified a variety of PFAS, such as the more commonly studied legacy PFAS, and newer PFAS such as PFBS and PFPeA, which may impact the findings. This review focused more on legacy PFAS due to the number of studies assessing their determinants, but this does highlight the need for further study of short-chain PFAS and PFAS alternatives in future studies evaluating PFAS determinants. Potential health outcomes associated with short-chain PFAS and alternatives are still not well understood, though short-chain PFAS appear to cross the placental barrier at rates like long-chain PFAS and limited evidence has suggested deleterious effects following exposure [175, 176]. Few studies evaluated PFAS mixtures as summed measures, such as evaluating summed PFSAs, summed PFCAs, and summed PFAAs [60, 77, 87, 93]. While human exposure usually includes a variety of PFAS, there has been limited effort to address the interactions and potential additive effects of PFAS mixtures and mixtures of additional environment chemicals. Several studies measured non-PFAS, such as polychlorinated biphenyls (PCBs) [61, 62, 67, 75, 77], organochlorines [including p,p’-dichlorodiphenyldichloroethylene (*p,p*’DDE), hexachlorobenzene (HCB), β-HCH, and 1,3,6,8-tetranitro carbazole (*t*-NC)] [61, 62, 67, 75], parabens [75], phthalates [62, 75], polybrominated diphenyl ethers (PBDEs) [61, 62, 77], phenols [including bisphenol A (BPA)] [62], and metals [62]. Future studies should evaluate potential effects of co-exposure to a variety of environmental contaminants.

The PFAS focused upon in this review—PFOA, PFOS, PFNA, and PFHxS—have several exposure sources, which can vary based on the specific PFAS. Exposure to these PFAS has been associated with residential water [177, 178], treated carpets [179], coated food materials [179], fish consumption [95], and more. Short-chain PFAS and alternative may not have the same exposure sources, or the same quantities seen in studies included in this review. Therefore, it cannot be assumed that similar findings will be seen in short-chain and alternative PFAS. As longer-chain PFAS are replaced by new chemicals, future study designs need better variables of exposure such as the changes suggested herein: accounting for PFAS with shorter elimination half-lives in humans, development of novel detection methods to quantify more PFAS, and introduction of statistical methods to evaluate PFAS mixtures in exposed individuals and communities [180].

The timing of sample collection can also impact findings, and the studies quantifying PFAS concentrations in pregnant mothers used a variety of timepoints for collection. This variability will be especially important when studying short-chain or rapidly excreted PFAS in future studies. Changes in the body throughout pregnancy may impact serum and plasma measurements and studies collected maternal blood samples at many different timepoints during pregnancy. Serum and plasma concentrations of PFAS can be impacted during pregnancy from dilution due to increased blood volume and transplacental transfer [181], so sample collection across pregnancy may result in differing PFAS concentrations. Glomerular filtration rate (GFR) increases after six weeks of gestation, which can accelerate PFAS excretion. Because plasma volume expansion and changes in GFR could be related to development in the fetus, these mechanisms could present noncausal associations between PFAS and determinants [182]. Sampling later into a pregnancy may also introduce confounding or reverse causality [183]. This potential source of exposure misclassification would likely be non-differential.

Age has been identified as a determinant of legacy PFAS concentrations in the general population [184, 185]; however, this may be due to the wider age range of participants in general population studies versus studies of pregnant mothers. As discussed previously, smoking and BMI as determinants of legacy PFAS concentrations have had mixed findings in the general population. Race and ethnicity have been identified as important determinants of PFOA, PFOS, and PFNA in the general population [115, 185], though findings have been mixed. Lastly, fish consumption has been associated with elevated PFOS concentrations in the general population [186–188]. As discussed previously, this may be due to changes in fish consumption habits prior to and during pregnancy based on past advice from medical professional and advisory bodies. Overall, our understanding of determinants of PFAS concentrations in all populations is lacking, though most findings from general studies appear like findings in pregnant mothers as presented in this review. Further study is needed to identify determinants of PFAS concentrations to identify habits and lifestyle patterns associated with increased exposure. A better understanding of exposure is critical to introducing policy to limit exposure, especially in vulnerable populations.Limitations of the studies reviewed include use of mainly self-reported factors, especially dietary habits and alcohol consumption, which may result in information bias. Potential misclassification would therefore likely be non-differential and bias associations towards the null. Additionally, most studies did not have information on potentially important indicators of PFAS exposure, such as the use of PFAS-containing personal care products, use of non-stick cookware, and occupational sources of exposure. Potential sources of exposure such as air or water pollution through proximity to a known point source of PFAS were not considered. Most studies did not quantify neonatal PFAS exposure, instead using maternal measurements. Transfer rates of PFAS across the placenta are not well understood, so fetal and neonatal exposure from pregnancy cannot be quantified. Lastly, many studies were cross-sectional in nature, which limits our ability to infer causality between presumed determinants and PFAS concentrations. Ideally, more longitudinal studies with repeated measurements through pregnancy paired with a measure at birth would establish temporality, limit the risk of exposure misclassification due to measurements at a single time point in pregnancy, and help us to better understand fetal exposure during pregnancy in future studies.

In utero exposure to persistent organic pollutants such as PFAS is associated with negative outcomes in pregnancy, after birth, and later in life [32]. PFAS have been associated with increased incidence of gestational diabetes, childhood obesity, preeclampsia, reduced lactation duration, and fetal growth restriction [189–196]. Unfortunately, the toxicological effects of PFAS as a mixture are generally unknown [197]. There is little understanding of potential interactions between analytes. A more thorough investigation of analyte interactions and measurement of more analytes in studies would aid in the understanding of determinants and outcomes related to these chemicals.

Within the published literature, there is an incomplete assessment of household factors and dietary habits, some of which were outlined in Tables 1 and 2, as they relate to predicting PFAS concentrations in pregnant mothers and neonates. Additionally, determinants were classified or measured differently across studies, making direct comparisons more complex. Utilizing similar, consistent categories for determinants in future research could address this issue. Many of the factors evaluated by studies in this review were included in only one to two studies and require further investigation (Table 1). 

## Conclusions

In this systematic review, we observed generally consistent evidence that parity, maternal nationality or country of origin, household income, and breastfeeding history predicted concentrations of certain PFAS in pregnant mothers and neonates. There was some evidence to suggest, fish consumption, maternal age, and maternal education may be determinants of maternal or neonatal PFAS concentrations. Maternal smoking history, pre-pregnancy BMI, and alcohol consumption did not have evidence to suggest they may be important determinants of maternal or neonatal legacy PFAS concentrations in pregnant mothers and their offspring. Differences in study design, study populations, or data collection methods could account for variability in findings across the reviewed articles. Given the potential risk of adverse outcomes in pregnant mothers and neonates exposed to PFAS, assessment of a variety factors–especially related to household characteristics and dietary habits–is necessary to identify more determinants of maternal and neonatal PFAS concentrations. Understanding groups of pregnant mothers and neonates at the highest risk of exposure allows for the implementation of appropriate public health interventions and the introduction of legislation where necessary. More focus should be directed towards short-chain PFAS and alternatives in future work as well as quantifying PFAS in neonates, where possible.

## Supplementary Information

**Additional file 1:** **Supplemental Table 1.** Descriptions of studies evaluating (*n*=35)predictors of PFAS concentrations in pregnant mothers or neonates.

## Data Availability

Data sharing is not applicable to this article as no datasets were generated or analyzed during the current study.

## Abbreviations

6:2 Cl-PFESA: 6:2 Chlorinated polyfluorinated ether sulfonate
BMI: Body mass index
BPA: Bisphenol A
CI: Confidence interval
Et-PFOSA-AcOH: 2-(N-Ethyl-perfluorooctane sulfonamido) acetic acid
ETS: Environmental tobacco smoke
FLEHS: Flanders Environmental and Health Study
GFR: Glomerular filtration rate
HCB: Hexachlorobenzene
HOME: Health Outcomes and Measures of the Environment
Me-PFOSA-AcOH: 2-(N-Methyl-perfluorooctane sulfonamido) acetic acid
N-alkyl FASA: N-alkyl perfluoroalkane sulfonamide
NHANES: National Health and Nutrition Examination Survey
NMeFOSAA: N-methyl perfluorooctanesulfonamidoacetic acid
PBDE: Polybrominated diphenyl ether
PBPK: Physiologically based pharmacokinetic
PC: Principal component
PCB: Polychlorinated biphenyl
PFAA: Perfluoroalkyl acid
PFAS: Per- and polyfluoroalkyl substances
PFBA: Perfluorobutanoate
PFBS: Perfluorobutanesulfonic acid
PFCA: Perfluoroalkyl carboxylic acid
PFDA: Perfluorodecanoic acid
PFDoA: Perfluorododecanoic acid
PFESA: Perfluoroalkyl ether sulfonic acid
PFHpA: Perfluoroheptanoic acid
PFHpS: Perfluoroheptanesulfonic acid
PFHxS: Perfluorohexane sulfonate
PFNA: Perfluorononate
PFOA: Perfluorooctanoate
PFOS: Perfluorooctane sulfonate
PFOSA: Perfluorooctanesulfonamide
PFPeA: Perfluoropentanoic acid
PFSA: Perfluoroalkane sulfonic acid
PFTrDA: Perfluorotridecanoic acid
PFUA: Perfluoroundecanoic acid
PFUnDA: Perfluoroundecanoic acid
POP: Persistent organic pollutant
PPAR: Peroxisome proliferator-activated receptor
p,p’-DDE: P,p’-dichlorodiphenyldichloroethylene
PRISMA: Preferred Reporting Items for Systematic Reviews and Meta-Analyses
SD: Standard deviation
SE: Standard error
SES: Socioeconomic status
*t*-NC: 1,3,6,8-Tetranitro carbazole
TTE: Transplacental transfer efficiency
WTC: World trade center
