# Supplementary material for: Determinants of maternal and neonatal PFAS concentrations: a review
Source: Environ Health. 2023 May 10;22:41. doi: 10.1186/s12940-023-00992-x (PMC10170754; doi:10.1186/s12940-023-00992-x)
Supplement: Supplementary file 1 — Additional file 1: Supplemental Table 1. Descriptions of studies evaluating (n=35)predictors of PFAS concentrations in pregnant mothers or neonates. [file 12940_2023_992_MOESM1_ESM.docx]

**Supplement material: Predictors of maternal and neonatal PFAS concentrations**

Supplemental Table 1. Descriptions of studies evaluating (n=35) predictors of PFAS concentrations in pregnant mothers or neonates.

| **Study** | **Design** | **Location (Time Period)** | **Population (n)** | **Maternal age** | **PFAS measured** | **PFAS matrix** | **Time of PFAS sample collection** | **Maternal or infant factor or predictor** | **Maternal or infant factor collection** | **Statistical analysis** | **Adjusted variables** | **Significant Results and Risk Estimates** | **Main findings** |
| --- | --- | --- | --- | --- | --- | --- | --- | --- | --- | --- | --- | --- | --- |

| Berg et al. 2014 | Longitudinal | Norway (2007-2009) | N=391 | -- | PFHxS  PFHpS  PFOS  FOSA  PFHpA  PFOA  PFNA  PFDA  PFUnDA  PFDoDA | Maternal serum | - collected around gestational week 20, 3 days after delivered, and 6 weeks after delivery | - Parity - Breastfeeding - Age - BMI - Diet (marine food, meat, salty snacks, berries, beef, tea, coffee) | Questionnaire | - Concentrations below LODs were replaced by LOD/√2 and only compounds with detection frequencies above 80% were evaluated in statistical models - Partial least square (PLS) regressions were used for data reduction and for selecting variables of specific interest, which were further studied using analysis of covariance (ANCOVA) - Study group was divided into three or four groups according to their consumption of the selected dietary variables - conducted a sensitivity analysis by removing six potential outliers and applied the same statistical methods on the reduced data set; overall results did not change substantially, and all samples were included in the final models | Varies by chemical model:   - PFHxS: sampling date - PFHpS: sampling date - PFOS: sampling date - PFOA: sampling date - PFNA: sampling date and age - PFDA: age and BMI - PFUnDA: age and BMI | - Parity was the strongest significant predictor for all the investigated PFASs, and nulliparous women had higher concentrations compared to multiparous women (10 ng/mL versus 4.5 ng/mL in median PFOS, respectively) - Serum concentrations of PFOS and PFOA of women recruited day 1–100 were 25% and 26% higher, respectively, compared to those women recruited in the last 167 days of the study (day 601–867), and the concentrations of PFNA, PFDA and PFUnDA increased with age - Dietary predictors explained 0–17% of the variation in concentrations for the different PFASs - Significantly elevated concentrations of PFOS, PFNA, PFDA and PFUnDA were found among high consumers of marine food. - The concentrations of PFHxS, PFHpS and PFNA were also increased in high consumers of game and elevated concentrations of PFHpS and PFOS were detected in high consumers of white meat - Study subjects with a high intake of salty snacks and beef had significantly higher concentrations of PFOA | - This study demonstrates that parity, sampling date, and birth year are the most important predictors for maternal PFAS concentrations. Dietary predictors of PFAS may vary in importance according to the compound |
| --- | --- | --- | --- | --- | --- | --- | --- | --- | --- | --- | --- | --- | --- |

| Bjerregaard-Olesen et al. 2016 | Cross-sectional | Denmark (2008-2013) | N=1438 | Median: 29 | PFHxS  PFOS  PFOA  PFNA  PFDA  **Σ**PFSA  ΣPFCA  ΣPFAA | Maternal serum samples | - first trimester (between weeks 11 and 13 of pregnancy) | - Age - Pre-pregnancy BMI - Previous miscarriages - Educational level - Country of birth - Smoking - Alcohol intake | Questionnaire | - For samples with concentrations below the LOD, replaced with LOD/2 - Only PFAS with levels above the LOD for at least 50% of samples included in analysis - Summed concentrations of three groups of PFAS - Log-transformed PFAS concentrations - Multivariable linear regression to compare PFAS and potential predictors - Determined confounders *a priori* | - Age - Pre-pregnancy BMI - Previous miscarriage - Education - Country of birth | - Total concentration of PFAAs was higher in older women - Normal weight women had a higher total concentration of PFAAs than underweight, overweight, and obese women - Higher levels were observed for women without previous miscarriages, women with a high educational level, women born in Denmark, non-smokers, and women who consumed alcohol before or during pregnancy - PFDA levels varied more across the categories of age, BMI, education, smoking, and alcohol consumption than any other PFAAs measured | - PFAS levels in nulliparous pregnant women differed across age, BMI, previous miscarriages, educational level, country of birth, smoking status, and drinking habits - The sum PFAS level was highest for women above 30 years of age with a normal BMI, high education, with no previous miscarriages, who were born in Denmark, consumed alcohol, and did not smoke |
| --- | --- | --- | --- | --- | --- | --- | --- | --- | --- | --- | --- | --- | --- |

| Brantsaeter et al. 2013 | Cross-sectional | Norway (1999-2008) | N=487 | -- | PFOS  PFOA  PFHxS  PFNA | Maternal plasma | - collected at time of enrollment (approximately 17 weeks gestation) | - Diet (shellfish, oily fish, lean fish, eggs, poultry, beef, pork, milk, bread, fruit, vegetable, oils, butter/margarine) - Maternal age - Marital status - Length of education - Urban vs rural residence - Smoking - Alcohol use - Type of housing - Native language - BMI - Parity - Pregnancy weight gain - Gestational week of blood draw - Marine fatty acids supplement use - Duration of breastfeeding - Time since most recent pregnancy | Questionnaires | - Results reported on complete case analysis - PFAS concentrations log-transformed - Dietary data explored with continuous variables as well as ranked categories - Determinants of PFAS concentrations identified separately for each chemical - Multiple regression models and used stepwise variable exclusion - Predictors were identified by optimizing multiple linear regression models using AIC | Varies by model:   - PFOS: nulliparity, time since most recent pregnancy, breastfeeding duration, maternal education, smoking, marital status - PFOA: nulliparity, time since most recent pregnancy, total breastfeeding duration, urban residence, household income, gestational weight gain - PFHxS: nulliparity, breastfeeding duration, education, BMI, urban residence - PFNA: nulliparity, time since most recent pregnancy, total breastfeeding duration, maternal education, marital status, maternal age at delivery, marine fatty acids supplement use | - Parity was the determinant with the largest influence on plasma PFAS concentrations, with r2 between 0.09 and 0.32 in simple regression models - In optimal multivariate models, when compared to nulliparous women, parous women had 46%, 70%, 19%, and 62% lower concentrations of PFOS, PFOA, PFHxS, and PFNA respectively (p<0.01) - Duration of breastfeeding associated with reduced PFAS levels in all models - PFOA showed the largest reduction from breastfeeding, with a 2–3% reduction per month of breastfeeding in typical cases - Levels of PFOS, PFOA, and PFNA increased with time since most recent pregnancy - Pregnancy-related factors were most important predictors; however, diet was a significant factor explaining up to 4% of the variance - One quartile increase in estimated dietary PFAS intake was associated with plasma PFOS, PFOA, PFHxS, and PFNA concentrations increases of 7.2%, 3.3%, 5.8% and 9.8% | - The history of previous pregnancies and breastfeeding were the most important determinant of PFAS in this sample - Time since most recent pregnancy also a predictor of PFOS, PFOA, and PFNA levels - Household income, education, urban residence, and marital status a predictor of some analytes - BMI, age, and smoking status also predictors of some analytes - Diet was significant   predictor explaining up to 4% of variance |
| --- | --- | --- | --- | --- | --- | --- | --- | --- | --- | --- | --- | --- | --- |

| Caron-Beaudoin et al. 2020 | Longitudinal (2004-2017) | Canada (2016-2017) | N=97 | Median: 24; Range: 16-40 | PFHxS  PFOS  PFOA  PFNA  PFDA  PFUdA | Maternal serum  Maternal plasma | - Combination of several biomonitoring efforts; varied by effort | - Diet (marine mammals, fish, mollusks, seaweeds, land mammals, wild birds, wild berries) | Questionnaire | - ½ LOD value was attributed to individual sample concentrations below the LOD - If more than 40% samples below LOD, contaminant not included in analysis - PFAS concentrations log-transformed - Analyses performed using a generalized linear model with an identity link - To investigate the proportion of exposure to PFAAs from country food consumption versus from consumer goods in pregnant women from Nunavik, the ratios of PFNA/PFOA, PFNA/PFOS, PFNA/PFHxS and PFUdA/PFDA were calculated using the geometric means of serum concentrations and compared to - Multiple linear regression models were used to determine if pregnancy and maternal characteristics and the consumption of country foods could explain the serum concentrations of PFAAs (individual congeners and sum of PFAAs) among pregnant women in 2016–2017 - Potential covariates chosen *a priori* - Sensitivity analyses: (1) including participant’s education (2) including house crowding conditions (3) including housing need of repair | - Maternal age - Number of previous pregnancies - Smoking status - Trimester of pregnancy | - Statistically-significant downward time trends were noted for concentrations of PFOS, PFOA and PFHxS in pregnant Nunavik women between 2004 and 2017 - Between 2011 and 2016-2017, PFNA, PFDA, and PFUdA maternal serum levels increased by 19, 13, and 21% - Among participants in 2016-2017, mean concentrations for PFNA, PFDA, and PFUdA were higher than those measured in women aged 18-40 years old in the Canadian Health Measure Survey (CHMS) - PFOA and PFHxS were lower than in CHMS, whereas PFBA, PFHxA, and PFBS were not detected in 2016-2017 - Ratios of serum/plasma levels of PFNA/PFOA, PFNA/PFOS, PFNA/PFHxS and PFUdA/PFDA were significantly higher in the 97 pregnant women from Nunavik recruited in 2016–2017 compared to CHMS - In multivariate models, PFHxS, PFOS, PFNA, PFDA and PFUdA levels in 2016–2017 were strongly associated with the omega-3/omega-6 PUFA ratio, indicating a positive association between marine country foods consumption and higher exposure to PFAAs | - PFHxS, PFOS, PFNA, PFDA, and PFUdA levels in 2016-2017 were strongly associated with the omega-3/omega-6 PUFA ratio, indicating a positive association between marine country foods consumption and higher exposure to PFAAs |
| --- | --- | --- | --- | --- | --- | --- | --- | --- | --- | --- | --- | --- | --- |

| Chang et al. 2021 | Cross-sectional | United States (2014-2018) | N=453 | Mean: 24.8 ; SD: 4.67 | PFHxS  PFOS  NMeFOSAA  PFPeA  PFOA  PFNA PFDA  PFUnDA | Maternal serum | - collection in “early pregnancy” | - Age - Education - Poverty income ratio - Marital status - Insurance status - Hospital - Sampling year - Parity - BMI - Tobacco - Alcohol - Marijuana - Drinking water source - Age of home - Distance to nearest industrial plant - Frequency of floor cleaning - Takeout consumption - Microwave popcorn consumption - Frequency of cosmetic product use - Frequency of lotion use - Frequency of hair products use - Frequency of shampoo | Questionnaire | - PFAS levels below the LODs were imputed with LOD/√2 in the descriptive analyses - Log-transformed PFAS concentrations - PFAS with low detection frequencies excluded from analysis - fitted the multivariable linear regression models to assess the associations between serum PFAS levels and potential predictors - Covariates selected a priori - Missing covariate information addressed through multiple imputations by chained equations with regression imputation approaches - The sensitivity analyses included: 1) the comparison between multiple imputations and complete case analysis for multivariate linear regression models to evaluate the impact of the missing values; 2) the comparison between simple imputation with LOD/√2 and multiple imputations using maximum likelihood estimation for PFAS values below LOD. - Multiple imputations presented because other methods biased effect estimates away from null | Sociodemographic predictors model:   - Age - Sampling year - Parity - BMI - Education - Poverty income ratio - Marital status - Insurance status - Hospital - Tobacco - Alcohol - Marijuana use   Home environment and behavioral predictors model:   - Education - Parity - BMI - Tobacco use - Marijuana use | - PFHxS, PFOS, PFOA, and PFNA were detected in >95% of the samples with PFOS having the highest concentrations - NMEFOSAA, PFPeA, PFDA, and PFUnDA were fofund in 40-50% of samples, whereas the detection frequencies for the 6 other PFAS were below 15% - When compared to NHANES participants matching sex, race, and age in this study, results showed similar concentrations of most PFAS, but higher concentrations of PFHxS - In adjusted models, education, sampling year, parity, BMI, tobacco and marijuana use, age of house, drinking water   source, and cosmetic use were significantly associated with serum PFAS concentrations | - In adjusted models, education, sampling year, parity, BMI, tobacco and marijuana use, age of house, drinking water source, and cosmetic use were significantly associated with serum PFAS concentrations |
| --- | --- | --- | --- | --- | --- | --- | --- | --- | --- | --- | --- | --- | --- |

| Colles et al. 2020 | Cross-sectional | Belgium (2008-2014) | N=529 dyads | -- | PFOS  PFBS  PFHxS  PFOA  PFNA | Cord plasma | - Collected shortly after birth by midwife | - Age - Pre-pregnancy BMI - Parity - Breastfeeding history - Food consumption habits (offal, eggs, fish, cheese, leafy vegetables, locally produced foods) - Use of personal care products - Education - Income - Seasonal trends - Alcohol consumption - Infant sex | Questionnaire | - Statistical analysis was carried out when at least 70% of the samples had exposure values above LOQ, values below LOQ were replaced by half the LOQ - Linear regression was used to identify those variables that significantly (p < 0.05) explained individual variability in exposure levels - PFAS concentrations log-transformed - Multiple linear regression was used to evaluate the effect of different variables on the biomarker level in the same model - Variables included in the models using a stepwise procedure and *a priori* selection | - Age - Pre-pregnancy BMI - Parity - Alcohol consumption before pregnancy - Duration of lactations - Sex of baby | FLEHS II (2007-2011)   - Duration of lactation negatively associated with PFOS [>6 months estimate: 0.67 (0.54-0.84)] and PFOA [>6 months estimate: 0.71 (0.59, 0.86)] - Consumption of local eggs and PFOS [estimate: 1.34 (1.17, 1.54)] - Consumption of local leafy vegetables [estimate: 1.25 (1.06, 1.48)] - Age of mother positively associated with PFOA [estimate >35 yrs: 1.49 (1.21-1.83)] - Multiparity negatively associated with PFOA [3+ child estimate: 0.79 (0.68-0.92)]   FLEHS III (2012-2015)   - Age of mother positively associated with PFOS [estimate >35 years: 1.79 (1.29-2.48)], PFOA [estimate >35 years: 1.39 (0.89-1.17)], PFHxS [estimate >35 years: 1.91 (1.41-2.59)], and PFNA [estimate >35 years: 1.84 (1.34-2.52)] - Duration of lactation negatively associated with PFOA [>6 months estimate: 0.67 (0.57-0.80)], PFHxS [>6 months estimate: 0.60 (0.48-0.74)], and PFNA [>6 months estimate: 0.64 (0.51-0.80)] - Multiparity negatively associated with PFOS [3+ child estimate: 0.73 (0.57-0.93)] - Alcohol consumption positively associated with PFOS [highest consumption estimate: 1.61 (1.28-2.01)], PFOA [highest consumption estimate: 1.41 (1.19-1.66)], PFHxS [highest consumption estimate 1.60 (1.30-1.97)], and PFNA [highest consumption estimate: 1.39 (1.12-1.74)] - Consumption of offal (yes vs no) PFOS [estimate: 1.60 (1.21-2.13)], PFOA [estimate: 1.71 (1.37-2.13)], and PFNA [estimate: 2.29 (1.71-3.08)] - Consumption of local produce negatively associated with PFOS [highest intake category estimate: 1.17 (0.96-1.43)] | - Lactation history, parity, maternal age and alcohol consumption during pregnancy were associated with most PFAS concentrations. Some dietary items may contribute to PFAS concentrations |
| --- | --- | --- | --- | --- | --- | --- | --- | --- | --- | --- | --- | --- | --- |

| Fabelova et al. 2023 | Cross-sectional | Europe (9 cohort studies: Spain, Norway, France, Belgium, Slovakia) Years ranging from 2003-2014 | N=5897 pregnant women  N=940 neonates | Mean: 30.2 | PFOS  PFOA  PFHxS  PFNA | Maternal serum or plasma  Cord serum or plasma | - Varies by cohort | - Age at delivery - Age at sampling - Parity - Previous breastfeeding - Pre-pregnancy BMI - Smoking history - Environmental tobacco smoke exposure - Sex - Gestational age - Maternal education - Paternal education - Food (seafood/fish, meat, offal, milk and dairy, eggs) | Questionnaire, clinic records | - Concentrations below LOQ were checked in each cohort separately and then imputed based on the percentage of values < LOQ in the cohort: by LOQ/√2 if <20% values were below LOQ or by LOQ/2 if ≥ 20% values were below LOQ - PFAS concentrations log-transformed - Associations between each variable and substance examined by linear regression and chi-square - Covariates chosen through DAGs to include in multiple linear regression; stepwise selection removed non-significant variables - Best model chosen based on adjusted r squared and AIC - K-fold cross-validation (k = 10) was applied as a data splitting method for model validation - he variable cohort was included as a fixed factor in the final models for all PFAS (except PFNA in newborns) - Sensitivity analysis performed by imputing model based on cohort and educational level of mother | - Varies by model | - Higher PFAS concentrations were associated with higher maternal age, primipara birth, and educational level, both for maternal blood and cord blood. - Higher PFAS concentrations in maternal blood were associated with higher consumption of fish and seafood, meat, offal and eggs. - In cord blood, higher PFHxS concentrations were associated with daily meat consumption and higher PFNA with offal consumption. - Daily milk and dairy consumption were associated with lower concentrations of PFAS in both, pregnant women and newborns. | - This study identified several determinants of PFAS exposure in pregnant women and newborns, including dietary factors, and these findings can be used for proposing measures to reduce PFAS exposure, particularly from dietary sources |
| --- | --- | --- | --- | --- | --- | --- | --- | --- | --- | --- | --- | --- | --- |
| Fisher et al. 2016 | Longitudinal | Canada (2008-2011) | N=1983 | Mean: 32.2 | PFHxS  PFOA  PFOS | Maternal plasma  cord plasma | - collected during first trimester - Cord blood collected at birth | - Parity - Maternal age - Income - Education - Smoking - Pre-pregnancy BMI - Food consumption (fish, bacon, hamburger, pork, poultry, and steak) - Foreign born - Fasting status - Sample collection year - Use of non-stick cooking vessels | Questionnaire | - Hypothesis tests were performed for contaminants with at least 50% of observations above LOD - Bonferroni-adjusted confidence intervals were calculated and used to identify the different groups when overall tests were significant - Prediction intervals were computed for geometric mean contaminant concentrations by the three demographic variables of interest, using Empirical Bayes estimates of the random effects - Observations below the LOD were imputed by LOD/2 | - Lipids | - There was a >90% detection of PFOA, PFOS, PFHxS, DDE, and PCB 138 and 153 in maternal plasma - Cord blood plasma had much lower detection rates - In a subset of 1^st^ and 3^rd^ trimester paired samples, PFAS concentrations were found to be strongly correlated - Parity, maternal age, income, education, smoking status, pre-pregnancy BMI and fish consumption were found to be significant predictors for most chemicals | - Parity, maternal age, smoking, household income, maternal education, sampling year, and cooking with non-stick cookware were associated with PFAS concentrations |

| Han et al. 2018 | Cross-sectional | China | N=369 | Mean: 28.35; SD: 4.06 | PFOA  PFOS  PFNA  PFDA  PFUA  PFHxS  PFBS  PFDoA  PFOSA  PFHpA | Maternal serum  Cord serum | - collected on admission for labor - collected immediately following delivery | - maternal age - paternal age - infant gender - maternal BMI - paternal BMI - dietary factors: milk, fish, egg, livestock, poultry, shellfish, fruits, and vegetables - drinking water source - gestational age - maternal smoking history - paternal smoking history - maternal alcohol use - paternal alcohol use - maternal education - paternal education - parity - household income | Questionnaire | - PFAS concentrations were log-transformed - For undetectable concentrations, LOD divided by the square   root of 2 was used   - Multiple stepwise linear regression models used to determine associations between related factors and cord serum PFAS - Covariates chosen *a priori* | -- | - Among the 10 PFAS, PFOA was the highest in maternal and cord serum - Maternal age, body mass index (BMI); smoking history; and intake of fish, milk, poultry, vegetables and tap water were   significantly related to PFASs concentrations in cord serum   - Twenty-seven (7.3%) HQ values exceeded 1 for both PFOA and the sum of PFOA and perfluorooctane sulfonate (PFOS), indicating potential concern for developmental toxicity in the local newborns | - Maternal age, body mass index (BMI); smoking history; and intake of fish, milk, poultry, vegetables and tap water were significantly related to PFASs concentrations in cord serum |
| --- | --- | --- | --- | --- | --- | --- | --- | --- | --- | --- | --- | --- | --- |
| Jia et al. 2023 | Cross-sectional | China (Jan-March 2022) | N=66 | -- | PFBS  PFBA  PFDS  PFHpA  PFDA  FHxSA  PFDoDA  PFHxA  PFHxS  PFNA  PFTeDA  PFUdA  PFOA  TFA  PFPeA  PFPrA  PFOS | Cord serum | Collected at birth | - Maternal education - Maternal age - Nutrient supplementation - Food intake (milk, fish, eggs, wheat, nuts) | Questionnaire; medical records | - When the concentration of PFASs was lower than the LOD, LOD/√2 was used - Log-transformed PFAS concentrations - Spearman's rank correlation analysis was used to detect the correlations between the PFASs and the content of each substance in cord serum - Kruskal-Wallis test was used to analyze the distribution of PFASs in cord serum among the different influencing factors | -- | - Higher education level, more significant the association with significantly higher levels of PFHxS, PFOA, and PFDA - PFDA concentrations increased with maternal age - Concentrations of most PFAS increased with fish consumption - Pregnant women supplemented with calcium regularly had a nearly 20-fold (0.0010–0.0195 ng·mL^-1^) reduction in serum PFDS concentrations compared to those who were never supplemented during pregnancy | - Maternal age, maternal education, diet, and nutritional supplementation during pregnancy can all affect umbilical serum exposure to PFASs |

| Kalloo et al. 2018 | Longitudinal | United States (2003-2006) | N=289 | -- | PFHxS  PFOS  PFOA  PFNA | Maternal serum | collected at 16 and 26 weeks gestation | - Maternal race - Marital status - Maternal education - Maternal age - Maternal BMI - Parity - Fish consumption - Fruit and veg consumption | Questionnaire | - imputed missing values among women who had at least one measured chemical concentration during pregnancy using the Markov Chain Monte Carlo (MCMC) method - generated 20 imputed data sets, averaged the imputed values, and used these values for all further analysis - Log-transformed PFAS concentrations - used k-means clustering to classify pregnant women into k clusters based on their chemical concentrations - Performed a principal components analysis - Used multinomial logistic regression and multivariable linear regression to identify predictors of cluster membership and principal component scores | - Maternal race - Marital status - Household income - Maternal educated - Maternal age at delivered - Maternal BMI - Parity - Fish consumption - Fruit and veg consumption | - black women had lower PC1 scores than white women [β = −0.78 (−1.22, −0.34)] - maternal BMI [β per SD increase in BMI = −0.48 (−0.64, −0.33)], parity [nulliparous vs multiparous β = 1.08 (0.77, 1.39)], and fresh fruits and vegetables consumption [consume daily vs less than daily β = 0.24 ( 0.07, 0.54)] were associated with PC1 scores - PC2 scores were inversely associated with women’s age [β = −0.78 (−1.22, −0.34)] - Being married vs unmarried [β = 0.75 (0.31, 1.19)] and each SD increase in age at delivery [β = 0.69 (0.52, 0.87)] were associated with higher PC3 scores - Parity associated with PC1 membership [β=1.08 (0.77, 1.39)] - consuming fruits and vegetables daily was associated with PC6 scores [β = −0.50 (−0.73, −0.27)] | - Cluster membership and PCA scores were associated with race, marital status, pre-pregnancy BMI, age, consumption of fresh fruits and vegetables, and parity |
| --- | --- | --- | --- | --- | --- | --- | --- | --- | --- | --- | --- | --- | --- |

| Kato et al. 2014 | Longitudinal | United States (2003-2006) | N=182 mothers 16-week; N=78 mothers delivery; N=202 cord serum; N=71 dyads | -- | Me-PFOSA-AcOH  PFHxS  PFOS  PFOA  PFNA | Maternal serum  Infant cord serum | - Collected at approximately 16 weeks gestation and at delivery - Collected at birth | - Maternal age - Education - Race - Household income - Gestational age - Parity - History of breastfeeding - Pre-pregnancy BMI - Serum cotinine | Questionnaire;  Biological samples | - PFAS concentrations log-transformed - Results below LOD substituted with LOD/√2 - Used non-parametric Wilcoxon signed rank test to compare each woman’s medians over the course of pregnancy and in infant cord sera - Used a paired t test to compare unadjusted GM for each pair of time points - Determined the Spearman rank correlations among the log_-_transformed concentrations of PFASs in maternal and cord sera at birth, and in maternal sera at birth and at 16 weeks gestation - Used linear regression to model the log-transformed PFAS of cord serum or mother’s serum at 16 weeks gestation for each predictor | Unadjusted | - The youngest category of maternal age (<25) was associated with lower GM PFOS [10.87 (9.16-12.9)] and PFHxS [1.02 (0.8-1.29)] - Black women had the lowest GM PFOS [10.85 (9.4-12.53)], PFOA [4.41 (3.82-5.1)], PFNA [0.73 (0.65-0.81)], and PFHxS [0.86 (0.72-1.03)] compared to white and other races - The lowest household income had lower GM PFOS [9.44 (7.73-11.51)] PFOA [4.1 (3.35-5.03)], PFNA [0.64 (0.55-0.74)], and PFHxS [0.84 (0.64-1.1)] - Obese women had lower GM PFOS [11.45 (9.77-13.43)] and PFNA [0.7 (0.63-0.79)] compared to normal and overweight women - Previous breast feeding associated with lower GM PFOS [11.85 (10.44-13.45)] and PFOA [4.47 (3.95-5.06)] - Education <12 years or =12 years lower GM PFHxS [0.94 (0.61-1.45), 1.14 (0.84-1.54)] - Active smokers had lower GM PFOS [9.04 (6.76-12.07)] vs secondhand and non-smokers - Parity >1 lower GM PFOS [11.37 (9.7-13.33)] and PFOA [4.83 (4.14-5.64)] vs parity=1 or 0 | - Women who were parous, with a history of previous breastfeeding, black, or in the lowest income category had significantly lower PFOS and PFOA GM concentrations than other women |
| --- | --- | --- | --- | --- | --- | --- | --- | --- | --- | --- | --- | --- | --- |

| Kingsley et al. 2018 | Longitudinal | United States (2003-2006) | N=468 | Mean: 29; SD: 5.8 | PFOA  PFOS  PFNA  PFHxS | Maternal serum    Cord serum | - Collected at 16 and 26 weeks of gestation - Collected within 48 hours of delivery | - Age - Household income - Serum cotinine - Race - Marital status - Parity - Fish consumption - Household income | Medical records;  Questionnaires;  Biological samples | - Log-transformed PFAS concentrations - Calculation Pearson correlation coefficients of each serum PFAS concentration between visits and between each of the PFAS at each visit - Used linear regression to examine association between gestational log-transformed serum PFAS and maternal factors - For all models, calculated the percent change in serum PFAS concentration for each predictor by exponentiating the regression coefficients, subtracting 1, and multiplying by 100 | - Maternal age at delivery - Maternal race - Parity - Household income - Serum cotinine concentrations   Child sex | - Household income associated with higher gestational serum PFAS concentrations, particularly PFOS - non-Hispanic black women had 18% (95% CI: −31, −3.8) and 43% (95% CI: −55, −29) lower serum PFOA and PFHxS concentrations, respectively, compared with white women - Women with one or more children had lower serum PFAS concentrations compared with primiparous women | - Income, race, and parity were associated with maternal PFAS concentrations |
| --- | --- | --- | --- | --- | --- | --- | --- | --- | --- | --- | --- | --- | --- |

| Lauritzen et al. 2016 | Cross-sectional | Norway and Sweden (1986-1988) | N=424 | Mean: 29; Range: 18-41 | PFOA  PFOS | Maternal serum | - Collected in second trimester | - Maternal age - Maternal height - Country of residence - Education - Maternal height, maternal weight, and maternal BMI - Smoking status - Alcohol consumption - Parity - Previous breastfeeding duration - Time since end of last breastfeeding period - GFR - Sampling date | Questionnaire | - PFAS concentrations log-transformed - Use multivariable linear regression to estimate associations between maternal characteristics and log-transformed serum PFAS - Calculated percent change in PFAS levels for each independent variable by exponentiating regression coefficients, subtracting 1 and multiplying by 100 - In sub-analyses investigate potential dose-response between smoking intensity and levels of PFOS | - Serum sampling date - Study site - Maternal age - Education level - Maternal BMI - Smoking status at conception - Alcohol consumption during pregnancy - Parity - Previous breastfeeding duration - Time since end of last breastfeeding period | - Previous breastfeeding duration, time since last breastfeeding period, sampling date, and country of residence were important factors associated with serum PFOS and PFOA - Smoking status and pre-pregnancy BMI were negatively associated with PFOS and maternal height negatively associated with PFOS and PFOA - GFR was negatively associated with PFOS in a sub-sample - Education level, pre-pregnancy BMI, and alcohol consumption varied in importance according to the compound under study | - Previous breastfeeding duration, time since last breastfeeding period, sampling date, and country of residence were important factors associated with serum PFOS and PFOA - Smoking status and pre-pregnancy BMI were negatively associated with PFOS - Maternal height borderline negatively associated with PFOS and PFOA   - GFR negatively associated with PFOS in a sub-sample |
| --- | --- | --- | --- | --- | --- | --- | --- | --- | --- | --- | --- | --- | --- |

| Lewin et al. 2017 | Longitudinal | Canada (2008-2011) | N=1983 | Mean: 32.2; SD: 5.1 | PFHxS  PFOA  PFOS | Maternal plasma | - collected during first trimester | - Maternal age - Household income - Parity - Smoking status - Pre-pregnancy BMI - Country of birth | Questionnaire | - Geometric mean blood concentrations for each chemical with 50% of the data above the LOD were calculated - Accounted for left censoring induced by values below the LOD using maximum likelihood estimation, Kaplan-Meier, and generalized Wilcoxon test - Linear models used to test variables’ of interest association with PFAS concentrations | Unadjusted | - Highest age category (≥35) associated with lowest GM PFHxS (0.91, p<.01) and PFOA (1.51, p<.01) - Parity >1 associated with lowest GM PFHxS (0.73, p<.01), PFOA (1.17, p<.01), and PFOS (3.51, p<.01) - Never smokers lowest GM PFHxS (0.99, p<.01) - Born in Canada highest GM PFHxS (1.11, p<.01) and PFOS (4.67, p<.01) - Highest household highest GM PFOA (1.75, p<.01) and PFOS (4.89, p<.01) | - Age, smoking, parity, country of origin, and household income were significantly associated with PFAS concentrations |
| --- | --- | --- | --- | --- | --- | --- | --- | --- | --- | --- | --- | --- | --- |

| Lien et al. 2013 | Cross-sectional | Taiwan (2004-2005) | N=439 mother-infant dyads | Median: 30.7; SD: 4.65 | PFOA  PFOS  PFNA  PFUA | Cord plasma | Collected at childbirth | - Delivery Method - Parity - Weight gain during pregnancy - Maternal age - Preterm delivery - Maternal education - Maternal nationality - Household income - ETS exposure during pregnancy - Maternal alcohol consumption - Use of cosmetics during pregnancy - Vitamin supplementation - Eating fish liver oil - Eating ocean fish oil - Calcium supplementation - Fish intake - Packing hot food in plastic bags - Silver dental fillings - Cockroaches in the home - Use of pesticides during pregnancy | Questionnaire | - PFAS concentrations log-transformed - T-tests and ANOVA to determine differences in PFAS levels among confounders - Statistically significant variables in unadjusted models or those reported as important variables in previous studies included in regression analysis | - Infant birth Weight - Infant gender - Maternal age - Preterm delivery - Delivery method - Parity - Weight gain during pregnancy - Maternal education - Maternal nationality - Annual household income - ETS exposure - Using cosmetics - Vitamin supplement - Fish intake - Packing hot food in plastic bags - Cockroaches in home | - The median concentrations for PFOA, PFOS, PFNA, and PFUA in cord blood were 1.86, 5.67, 3.00, and 13.5 ng/mL , respectively - The median concentrations for PFOA, PFOS, PFNA, and PFUA in cord blood were 1.86, 5.67, 3.00, and 13.5 ng/mL , respectively - After adjusting for potential confounders, multiple linear regression models revealed that log10-PFOA was positively associated with maternal age (b = 0.011) and negatively associated with multiparity (b = -0.044). Log10-PFOS was negatively correlated with birth weight (b = -0.011) and higher maternal education (senior high school: b = -0.067; university: b = -0.088) - Log10-PFUA tended to negatively associate with gender, male infants (b = -0.075), and using cosmetics during pregnancy (b = -0.065) - presence of cockroaches in the home was positively associated with log10-PFOA (b = 0.041) and 1og10-PFNA (b = 0.123) | - In adjusted analyses, maternal age positively predicted PFOA - Cockroaches in the home was positively associated with PFOA and PFNA - Parity was negatively associated with PFOA, higher maternal education, and use of cosmetics during pregnancy - Male infants were associated with lower PFUA compared to females |
| --- | --- | --- | --- | --- | --- | --- | --- | --- | --- | --- | --- | --- | --- |

| Long et al. 2015 | Cross-sectional | Greenland (2010-2013) | N=207 | Mean: 27.5; SD: 5.2 | PFOS  PFHxS  PFOA  PFNA  PFDA  PFUnA | Maternal serum | - collected at inclusion into the study (mean gestational age 25.1 weeks, range 7-40 weeks) | - Food intake (marine mammals, seabirds, fish, terrestrial species, imported food - Heavy metal concentrations - Region of household | Questionnaire; blood sample collection | - Grouped PFAS in PFSA and PFCA - PFAS concentrations log-transformed - Used one-way ANOVA to compare the continuous variables and PFAS. If significant, ad hoc tests performed - Test for equal variances of variables using Levene's test - Comparisons of means for chemicals among regions performed by General Linear Model adjusted for age and n − 3/n − 6 ratio - Spearman’s correlation to assess bivariate correlation of identified components, single PFAS congeners, heavy metals with lifestyle factors - Used multiple linear regression to assess relationship between serum PFAS and maternal factors | - Age - n − 3/n – 6 (fatty acids) - Region | - Significant regional differeneces for PFOS, PFHxS, and PFHpS with median levels of 10.15, 0.70, and 0.19 ng/mL, respectively - PFUnA, PFNA, and PFDA differed significantly among regions - Significantly higher levels of PFOS, PFHxS, PFHpS, PFUnA, PFNA, and PFDA in the Inuit women from North and East compared to South and West regions - ∑PFSA and the heavy metal Hg and trace element Se significantly positive in the North region - Trace element se positively associated with ∑PFAS in the West region and data pooled for all regions | - In the North region, PFAS were associated with both selenium and mercury - PFAS concentrations were associated with region |
| --- | --- | --- | --- | --- | --- | --- | --- | --- | --- | --- | --- | --- | --- |

| Makey et al. 2017 | Cross-sectional | Canada (2007-2008) | N=152 mothers | Mean: 33.9; Range: 25.5-41 | PFOS  PFOA  PFNA | Maternal serum | Mean sample collection: 14.8 weeks (±0.7, 12.3–17.1) | Airborne precursors | Air samples | - Results below the LOD substituted with LOD/√2 - PFAS concentrations not log transformed - Spearman’s correlation coefficients to examine relationships between chemicals in dust and air - Confounders determined *a priori* and using a 10% change in the beta coefficient - PrePFAA concentrations modeled as tertiles - Sensitivity analysis: (1) created a summed exposure metric for each precursor group (2) for relationships between environmental preFAAs and serum PFAAs with potential influential data points, performed regression analysis with and without extreme observations | - Parity | - PFNA in air and PFNA in serum [β (95% CI) = 1.1 (0.35, 1.9), p=0.002] - PFNA in dust and PFNA in serum [β (95% CI) = 1.1 (0.38, 1.8), p=0.003] | - PFNA in air and vacuum dust predicted serum PFNA |
| --- | --- | --- | --- | --- | --- | --- | --- | --- | --- | --- | --- | --- | --- |

| Manzano-Salgado et al. 2016 | Cross-sectional | Spain (2003-2008) | N=1216 | -- | PFHxS  PFOS  PFOA  PFNA | Maternal plasma | - Collected at approximately 12 weeks of pregnancy | - Country of birth - Region of residence - Type of residence zone - Education - Social class - Parity - Age - Breastfeeding history - Pre-pregnancy BMI - Smoking at beginning of pregnancy - Dietary factors: total fish and shellfish, total meats, eggs, dairies, cereals and pasta, fruits and vegetables, vegetable oil, and tap water | Questionniare | - Replaced maternal PFAS concentrations under the LOD with LOD/2 - Log-transformed PFAS concentrations - Used generalized additive models to examine the linearity of the relationship between each PFAS and the socio-demographic characteristic and food groups - Used multivariable linear regression models to examine the relationship between maternal factors and PFAS concentrations - Used ANOVA models to evaluate the contribution of the covariates to the overall variability of PFAS concentrations | - Maternal country of birth - Region of residence - Age - Previous breastfeeding - Parity - Pre-pregnancy BMI | - GM PFAS concentrations ranged from 0.55 ng/mL for PFHxS to 5.77 ng/mL for PFOS - Women born outside of Spain had lower PFAS concentrations (e.g. GM ratio for PFHxS 0.53[95%CI: 0.46, 0.60] than Spanish women. PFHxS and PFOA concentrations were higher in mothers from the regions of Sabadell (2.13[1.93, 2.35] and 1.73[1.60, 1.88], respectively) and Valencia (1.40[1.28, 1.54] and 1.42[1.31, 1.53], respectively) than Gipuzkoa - PFOA and PFNA concentrations decreased with parity (≥2 children: 0.79[0.67, 0.94] and 0.82[0.68, 0.99], respectively). Younger women (i.e. b25 years) had lower PFHxS (0.73[0.62, 0.86]) and PFOS (0.85[0.75, 0.96]) concentrations than older women - PFHxS and PFOA concentrations were lower in women who previously breastfed for N6 months compared to those who never breastfed (0.79[0.67, 0.94] and 0.82[0.71, 0.95], respectively) - High intake of fish and shellfish during pregnancy (i.e. ≥5.6 servings/week) was associated with 11% (1.11[1.04, 1.18]) higher PFOS concentrations than the lowest intake group - ANOVA models explained 26% to 40% of PFAS concentrations variability | - Prenatal PFAS concentrations were mainly determined by maternal country of birth, region of residence, previous breastfeeding, and age. - Fish and shellfish intake also contributed to PFOS and PFOA concentrations |
| --- | --- | --- | --- | --- | --- | --- | --- | --- | --- | --- | --- | --- | --- |

| Marks et al. 2021 | Cross-sectional | Norway (MoBa1999-2008), United Kingdom (ALSPAC 1991-1992) | MoBa n=276; ALSPAC n=422 | -- | PFOA  PFOS  PFHxS  PFNA | Maternal plasma/serum | MoBa: mean collection week 18 pregnancy  ALSPAC median collection week 15 pregnancy | - Foods (sausages, burgers, pies, pasties, meat, poultry, liver, white fish, other fish, shellfish, eggs, quiche, cheese, pizza, chips, roast potatoes, rice, pasta, potato crisps, baked beans, peas, green leafy vegetables, carrots, root vegetables, salad, fresh fruit, pure juice, pudding, oat cereals, wholegrain cereals, cakes/buns, crisps, biscuits, chocolate bars, pulses, nuts, chocolate, sweets) | Questionnaire | - ALSPAC coded each food item according to frequency of weekly consumption - Converted MoBa frequencies to weekly - Reduced rank regression (RRR) applied to extract dietary patterns from 38 food groups, specifying measured concentrations of persistent EDCs in blood as the response variables, which were grouped by class (PFAS, PCBs, and OCPs) - Food groups with factor loading values ≥0.2 or ≤−0.2 were considered the principal contributors to each derived dietary pattern; a high absolute value of a factor loading indicates higher contribution of that food group to the dietary pattern - Using RRR, a pattern score for each dietary pattern was calculated as a continuous measure for each individual - Outcomes of these models included blood concentrations of individual EDCs and totals by class (PFAS, PCBs, and OCPs), calculated as the summed total of Z scores within a class | - Maternal race/nativity - Maternal age - Maternal education - Pre-pregnancy BMI - Parity - Smoking during pregnancy - Gestational age at sample collection - Total energy intake | - Within ALSPAC, all patterns (PFAS, PCB, and OCP) were characterized by high consumption of meat, poultry, white fish, and biscuits - In MoBa, high consumption of sausages and burgers (representing processed meats), pasta, and chocolate bars characterized PCB and OCP dietary patterns, while high consumption of cheese characterized the PFAS pattern - Across both cohorts, PFAS patterns were characterized by high consumption of cheese | - Dietary pattens high in animal-based products appear to be associated with persistent EDC concentrations among pregnant women |
| --- | --- | --- | --- | --- | --- | --- | --- | --- | --- | --- | --- | --- | --- |
| Mehta et al. 2020 | Cross-sectional | United States (2011-2013) | N=98 | -- | PFHxS  PFOS  PFDeA  PFNA  PFOA | Maternal serum | - collected at baseline between 10 and 24 weeks | - Gestational age - Pre-pregnancy BMI - Parity - Healthy eating index - Race/ethnicity - Poverty status - Education - Marital status - Food insecurity - Smoking status - Lipid concentrations | Questionnaire; medical records | - Relationship between factors and chemical concentrations were initially examined with bivariate linear regression models - Retained variables were fit into a model and backwards elimination used - Effect modification by income or race/ethnicity tested - Stratified by poverty status - Used three methods of grouping chemicals: hierarchical clustering, a chemical burden sum score approach, and PCA - Log-transformed PFAS values for each individual chemical with a ≥50% DF - Linear regression analysis with backward selection used to determine if participant characteristics were associated with each principal component | Unadjusted | - Food insecurity was positively associated with PFOS [β=0.27 (0.04, 0.50), p=0.02] | - Food insecurity was positively associated with PFOS - No other characteristic were associated with PFAS analytes in final and exploratory models |

| Ode et al. 2013 | Cross-sectional | Sweden (1978-2001) | N=237 dyads | -- | PFOS  PFOA  PFNA | Maternal serum samples  Cord serum samples | - Both samples collected at delivery | - Maternal age - Parity - BMI - Smoking habits - Country of origin - Gestational duration - Newborn sex | Swedish Medical Birth Register | - Spearman’s rank correlation used to explore correlations between PFAS and determinants - Wilcoxon signed-rank test used to compare the levels between maternal and cord serum - Mann-Whitney U test and Kruskal-Wallis test used to determine if significant differences between groups - PFAS concentrations below the LOD replaced with the value 0.20ng/ml - Association between year of sampling and measured PFAS levels tested for significant with linear regression | -- | - Country of origin a predictor of PFAS, with women originating from Nordic countries having highest levels and the lowest in women from the Middle East, North Africa, and sub-Saharan Africa - Multiparous women had   lower serum PFOA levels (1.7ng/ml) than primiparous women (2.4ng/ml)   - Umbilical cord serum PFAS concentrations showed roughly similar patterns as the maternal samples - PFOS levels increased during the study period in native Swedish women | - PFOS levels tended to increase while PFOA and PFNA levels were unchanged between 1978 and 2001 in the study population - Maternal country of origin, parity, and gestational duration may be associated with PFAS exposure |
| --- | --- | --- | --- | --- | --- | --- | --- | --- | --- | --- | --- | --- | --- |

| Papadopoulou et al. 2016 | Longitudinal | Norway (2003-2009) | N=100 | -- | PFOS  PFOA  PFHxS  PFNA  PFUnDA  PFDA | Maternal plasma | - collected weeks 17-18 gestation | - Breastfeeding history - Fish consumption - Year of delivery - Time between pregnancies - Maternal GFR | Questionnaire;  Biological sample | - PFAS concentrations log-transformed - Performed multiple linear regression analysis to estimate the relative change in PFAS concentrations of the 2^nd^ pregnancy according to maternal characteristic - women were sub-categorized according to exclusivity of breastfeeding (exclusive breastfeeding at 6-8 months for duration of at least 10 months: 16 of 24 women; for duration of more than 10 months: 21 of 27 women) - tested whether fatty fish, lean fish, fish liver or shellfish consumption (in times/week) was related to PFASs concentrations in the 2nd pregnancy for 76 women who reported their fish intake | - Maternal age at 2^nd^ delivery - Year of 2^nd^ delivery - Time between pregnancies - Breastfeeding between pregnancies | - A 10% increase in PFAS in the 1st pregnancy associated with an increase in the 2nd pregnancy of 9.0% for PFOS, 7.2% for PFOA, 14.2% for PFHxS, 6.1% for PFNA, 9.8% for PFUnDA, and 6.9% for PFDA - Regarding the year of the 2nd delivery, maternal PFOA concentrations declined by 8.7% for every year increase - Breastfeeding between pregnancies accounted for almost as much variation in PFOA in the 2nd pregnancy (29%) as did the PFOA concentration in the 1st pregnancy (34%) - Time between pregnancies an important determinant for PFNA and PFDA; observed a 31% and 75% increase for every year increase in the time between pregnancies. - Maternal GFR was a predictor of PFUnDA concentration for the 86 women with available information - intake of shellfish but not other fish was an important predictor of PFUnDA | - The timing of previous pregnancies, breastfeeding, intake of shellfish, and maternal GFR can have substantial effects on PFAS concentrations |
| --- | --- | --- | --- | --- | --- | --- | --- | --- | --- | --- | --- | --- | --- |

| Richterová et al. 2018 | Longitudinal | Slovakia (2010-2012) | N=322 | Mean: 28.7; SD: 4.7 | PFOA  PFOS  PFNA  PFHxS  PFBA  PFPeA  PFHxA  PFHpA  PFDA  PFUdA  PFDoA  PFTrDA  PFTeDA  PFBS  PFDS  PFOSA  NMeFOSA  NEtFOSA | Infant cord serum | - Collected at birth | - Maternal age - Parity - Marital status - Education - Employment - Pre-pregnancy BMI - Weight gain during pregnancy - Smoking - Alcohol consumption - Fish consumption | Questionnaire | - Only PFASs with >80% of samples >LOD were included in further analyses (namely PFOS, PFHxS, PFOA, and PFNA) - PFASs concentrations <LOD were imputed using LOD/√2 - PFAS concentrations log-transformed - Multivariable linear models, included covariates based on literature and results from bivariate regression - Employment was strongly associated with parity, maternal age, and education and not included in final model - Strong predictors for fast food consumption were parity and maternal age and therefore not included in the final model | - Age at delivery - Parity - Education - Fish consumption | - Older age associated with greater PFNA [β=1.27 (1.04, 1.55)] and PFHxS [β=1.30 (1.06, 1.60)] - Primiparity associated with higher PFOA [β=1.49 (1.18, 1.89)], PFOS [β=1.25 (1.03, 1.53)], PFNA [β=1.30 (1.05, 1.60)], and PFHxS [β=1.49 (1.20, 1.86)] - Higher education associated with higher PFNA [β=1.32 (1.04, 1.68)] - Fish consumption associated with greater PFNA [β=0.49 (0.26, 0.92) | - Parity was the main determinant of PFAS exposure - Maternal age, educational level, and fish consumption were also predictors of PFAS concentrations - No association between PFAS cord blood levels and BMI, smoking or drinking alcohol before pregnancy was found |
| --- | --- | --- | --- | --- | --- | --- | --- | --- | --- | --- | --- | --- | --- |

| Rush et al. 2018 | Cross-sectional | Norway (1999-2008) | N=1090 | -- | PFOA  PFNA  PFDA  PFUnDA  PFHxS  PFHpS  PFOS | Maternal plasma | - Collected approximately 17-18 weeks gestation | - Oral contraceptive use - Recency of oral contraceptive use - Duration of oral contraceptive use - Age at first oral contraceptive use | Questionnaire | - Plasma PFAS concentrations <LOQ were quantitated and reported when possible - Linear regression analyses were used to estimate the association between plasma PFAS concentrations and OC use in the previous 12 months, separately for each PFAS - PFAS concentrations were log-transformed - Covariates included in model if covariate was statistically significantly associated   with the PFAS or its inclusion in the age-adjusted model changed effect estimates by 10% or more   - Tests of interaction were conducted by including dummy variables | - Education - Menstrual length cycle - parity | - Except for PFDA and PFUnDA, women who used OCs in the 12 months preceding the baseline interview had 12.9-35.7% higher PFAS concentrations than never OC users - Past OC use positively associated with PFAS (estimates ranged 7.2-32.1%) - Compared with never users, using OCs for 10+ years associated with increased PFAS, except for PFDA and PFUnDA (estimates ranged 18.9-46.2%) | - Characteristics of OC use and duration of its use, may be important considerations when investigating relationships between women’s reproductive outcomes and PFAS |
| --- | --- | --- | --- | --- | --- | --- | --- | --- | --- | --- | --- | --- | --- |

| Sagiv et al. 2015 | Longitudinal  Longitudinal | United States (1999-2003) | N=1668 | -- | PFOS  PFOA  PFHxS  PFNA  Et-PFOSA-AcOH  Me-PFOSA-AcOH | Maternal plasma | - collected during “early pregnancy” (median=9.7 weeks gestation; range=4.8-21.4 weeks) | - Maternal age - Marital status - Race/ethnicity - Smoking status - Education - Household income - Parity - Pre-pregnancy BMI - Breastfeeding history - Plasma albumin - GFR - Year of enrollment - Gestational age at blood draw | Questionnaire | - Estimates PFAS geometric means to account for the skewed distribution of PFAS concentrations in the population, and estimated unadjusted partial sum of square *p*-values (*p*-value across multiple categories) for each predictor using linear regression models - Fitted linear regression models to generate adjusted estimates and 95% confidence intervals (CI) for predictors - Log-transformed PFAS concentrations   calculated percent change in PFAS concentration for each predictor by exponentiating regression coefficients, subtracting 1 and multiplying by 100 | - Age - Race/ethnicity - Maternal education - Paternal education - Marital status - Household income - Smoking status - Year of enrollment - Parity - Breastfeeding history - Pre-pregnancy BMI - Gestational age at blood draw - GFR - Plasma albumin | - PFAS concentrations were lower with age, except for PFNA (3.7% change per 5 years) - PFAS concentrations lower with educational attainment; associations weaker for PFNA and for PFHxS - lower PFOS, PFOA and PFNA concentrations at the lowest income level (<40k/year) vs. the highest (>70k/year) - Except PFNA, all PFAS concentrations declined over the enrollment period, most strongly for Et-PFOSA-AcOH and Me-PFOSA-AcOH - Fully adjusted models showed lower PFAS among nulliparous vs. parous women, though opposite trend for Et-PFOSA-AcOH and Me-PFOSA-AcOH - All PFAS higher among women who never breastfed, especially PFOS and PFOA - PFAS also higher for mothers with higher pre-pregnancy BMI - PFAS inversely associated with GFR and positively associated with plasma albumin | - Higher early pregnancy PFAS concentrations were associated with younger age (except PFNA), less educational attainment, nulliparity, no history of breastfeeding and higher pre-pregnancy body mass index in adjusted models - lower GFR was associated with 3–4% higher PFAS concentrations and higher albumin was associated with 4–6% higher PFAS concentrations - results show associations consistent (parity and breastfeeding) and less consistent (age and education) with previous studies |
| --- | --- | --- | --- | --- | --- | --- | --- | --- | --- | --- | --- | --- | --- |

| Santos et al. 2021 | Cross-sectional | Brazil | N=135 dyads | -- | PFOA  PFOS | Maternal serum  Cord serum | - collected between 28 and 32 weeks - collected at birth | - maternal age - income - ethnicity - education - parity - pre-pregnancy BMI - Dietary factors: fish, seafood, vegetables, fruit, meat, eggs, processed meat, packaged meals - Alcohol consumption - Smoking history - Employment status - Drinking water - Home renovations - Infant sex | Questionnaire | - PFAS concentrations were log-transformed - Performed ANOVA and t-tests for PFOA and PFOS concentrations according to maternal characteristics - Used multivariable linear regression models to examine associations between potential predictors and PFOA and PFOS - Stepwise approach used with AIC to define the optimal set of variables | - Education - Income - Race - Pre-gestational BMI - Smoking - Alcohol consumption - Gestational age - Primiparity - Age - Fish consumption | - Age negatively associated with PFOA [age 16-19 vs 40+ % difference: 437.0 (79.9, 1503.2)] - Fish consumption positively associated with PFOA [0.6-2.0 servings/wk vs 2.1+ servings/wk % difference: -48.2 (-70.0, -10.7)] - Number of rooms in home associated with PFOS [up to 4 vs above 4 % difference: 60.7 (6.4, 142.7)] - Tobacco use associated PFOS [yes vs no % difference: -35.6 (-58.1, -0.9)] | - In this study, age, fish consumption, passive smoking, and the number of rooms in the house were predictors of maternal PFAS levels |
| --- | --- | --- | --- | --- | --- | --- | --- | --- | --- | --- | --- | --- | --- |

| Singer et al. 2018 | Cross-sectional | Norway (1999-2008) | N=1936 | -- | PFOA  PFNA  PFDA  PFUnDA  PFHxS  PFHpS  PFOS | Maternal plasma | collected between 17-18 weeks gestation | - Menstrual cycle irregularity - Menstrual cycle length | Questionnaire | - Examined the distribution of participant characteristics by irregular menstrual cycles and by categories of menstrual cycle length - examined the distribution of PFAS levels two ways: (1) restricted to PFAS values above the LOQ and (2) including PFAS values above the LOQ and measured values below the LOQ - Conducted multiple linear regression to examine association between menstrual cycle characteristic and PFAS concentrations - Fit separate models for each PFAS - Log-transformed PFAS concentrations - Selected covariates *a priori* - Stratified by parity and use of oral contraceptives | - Maternal age - Pre-pregnancy BMI - Smoking status - Educational level - Gross income - Parity - Use of oral contraceptives in the past 12 months - Months between previous pregnancy and current pregnancy - Months of breastfeeding following most recent live-birth - Sampling group status | - Irregular cycles were not associated with PFAS concentrations - Overall, we found no evidence of associations between menstrual cycle length and PFAS concentrations - In subgroup analyses we found some evidence, among parous women, of decreased PFHpS and PFOS with short menstrual cycles; we also found, among recent OC users (in the 12 months before the questionnaire) increased PFNA and PFUnDA with long cycle length | - There was little evidence of menstrual cycle characteristic as determinant of PFAS concentrations; however, they did observe some associations between cycle length and PFAS concentrations with some select PFAS compounds in subgroup analyses |
| --- | --- | --- | --- | --- | --- | --- | --- | --- | --- | --- | --- | --- | --- |

| Shu et al. 2018 | Longitudinal | Sweden (2007-2010) | N=1616 | Mean: 31; SD: 4.7 | PFNA  PFDA  PFUnDA  PFDoDA  PFHxS  PFHpA  PFOA  PFOS | Maternal serum | - collected between weeks 3 and 27 of pregnancy (median week was 10 weeks where 96.1% of the samples were collected before week 13) | - Parity - Fish consumption - Living location - Cotinine/smoking | Questionnaire;  Birth Registry;  Biological samples | - PFAS concentrations were log-transformed - Pearson correlation test used to explore unadjusted relationship between serum PFAA levels and age, and the Spearman correlation test was used for univariate analyses of PFAA levels in relation to the fish intake index - Covariates were included in multivariable models if they were significantly associated with four or more serum PFAS concentrations - Least square geometric means of PFAS concentrations by sampling year were calculated - Percent changes in PFAS concentrations by sampling year were calculated | - Parity - Maternal age - Fish intake - Smoking | - Primi and multiparous women had lower levels of PFAS in serum for PFNA, PFDA, PFDoDA, PFUnDa, PFHxS, PFHpA, PFOA, and PFOS - Mother’s age significantly associated with PFDoDA (positive), PFUnDA (positive), and PFOA (smoking) - Smoking significantly association with PFDA (negative), PFDoDA (negative), PFUnDA (negative), and PFHxS   (positive)   - Fish intake positively associated with PFNA, PFDA, PFDoDA, and PFOS | - Parity, higher fish intake, smoking, and maternal age are determinants for serum levels of PFAS in pregnant women |
| --- | --- | --- | --- | --- | --- | --- | --- | --- | --- | --- | --- | --- | --- |

| Spratlen et al. 2019 | Longitudinal | United States (2001-2002) | N=279 (n=120 exposed to WTC, n=159 reference group) | Median: 31.0; IQR: (27.3-34.6) | PFOS  PFOA  PFHxS  PFDS  PFNA | Maternal plasma  Cord plasma | - collected at delivery - collected at delivery | Exposure to disaster through work or residence | Questionnaire | - PFAS assessment was restricted to compounds quantified in ≥50% of samples (PFOS, PFOA, PFNA, PFHxS and PFDS) - To account for differences in maternal plasma vs cord blood samples, used 78 paired cord blood and maternal plasma samples from the HOME study to create cord blood concentration predictions - Separate prediction models run for PFOS, PFOA, PFNA, and PFHxS - PFAS concentrations log-transformed - samples <LOQ were imputed as the LOQ divided by √2 - Multivariable models to assess association between WTC exposure and PFAS concentrations - Covariates included in the model determined *a priori* - Sensitivity analyses: (1) ran analyses using just PFAS concentrations measured in cord blood to evaluate consistency with analyses using maternal-to-cord transformed concentrations (2) checked whether using a complete case analysis biased results using MICE (3) among a subset of participants with information on local fish intake, ran analyses additionally adjusting for this variable to understand whether other sites of contamination might confound associations | - Child sex - Maternal age - BMI - Education - Marital status - Parity - Trimester pregnant during the 9/11 disaster - Home smoking exposure - Maternal race | - Living/working within two miles of WTC was associated with 13% higher PFOA concentrations compared with the reference group [GMR (95% CI): 1.13 (1.01, 1.27)] - The association was stronger when comparing only those who lived within two miles of WTC to the reference group [GMR (95% CI): 1.17 (1.03, 1.33)], regardless of work location | - These results provide evidence that exposure to the WTC disaster during pregnancy resulted in increased PFAS concentrations, specifically PFOA |
| --- | --- | --- | --- | --- | --- | --- | --- | --- | --- | --- | --- | --- | --- |

| Tian et al. 2018 | Cross-sectional | China (2012) | N=981 | Mean: 27.82; SD: 3.48 | PFHxS  PFOS  PFOA  PFNA  PFDA  PFUdA  PFDoA  PFTrDA | Maternal plasma | - Collected between 12-16 weeks gestation | - Maternal age - Household income - Educational level - Pre-pregnancy BMI - Parity - Smoking status - Living rooms decorated within the past two years before enrollment - Residential area - Dietary habits (red meat, animal offal, poultry, fish, eggs, pastries, fried food, wheat, coarse cereals, tubers, soy products, puffed food, nuts) - Drinking water source - Exposure to environmental contaminants (organic solvents, paints, pesticides, and heavy metals) | Questionnaire | - PFAS with a detection rate of 80% or above included in analysis - PFAS - concentrations below the LODs were replaced with LOD/√2 - PFAS concentrations log-transformed - Pearson correlation to examine correlations after log transformation - ANOVA and Student’s t-test were used to compare GMs of PFAS concentrations across factors - Multivariate linear models were used to examine association between PFAS concentrations and maternal factors - Confounders determined a priori - VIFs of each variable used for collinearity diagnostics | - Age - Education - Income - Parity - Pre-pregnancy BMI - Smoking status - Residence in rooms decorated within last two years | - PFHxS concentration in pregnant women being overweight (BMI ≥ 25 kg/m2) was 0.85-fold (95% CI: 0.75–0.98) those in underweight women (BMI < 18.5 kg/m2) - Pregnant women of 25–30 years and ≥30 years of age had between 1.11-fold (95% CI: 1.00–1.24) and 1.51-fold (95% CI: 1.21–1.89) higher concentrations of most long-chain PFASs than those of < 25 years of age - PFOA, PFNA, PFDA, and ∑PFASs in pregnant women with per capita household incomes ≥8000 CNY were between 0.86- fold (95% CI: 0.76–0.97) and 0.90-fold (95% CI: 0.82–0.98) those of   women with per capita household incomes < 4000 CNY   - Women with college education had higher concentrations than those with education levels below high school (GM ratio ranged from 1.19 to 1.28, p<.05) - For PFNA, women with education at college level or above or at high school level had 1.17-fold (95% CI: 1.02–1.34) and 1.22-fold (95% CI: 1.09–1.46) higher concentration than those below high school level - Multiparous women had between 1.11-fold (95% CI: 1.03–1.20) and 1.22-fold (95% CI: 1.07–1.39) higher   concentrations of PFOS, PFNA, PFDA, PFUdA and ∑PFASs than | - Pregnant women who were older, multiparous, well educated, passive smokers, with lower per capita household incomes, and had lived in rooms decorated within the past two years had higher PFAS concentrations, after mutual adjustment for maternal sociodemographic characteristics and lifestyles - Regarding dietary factors, intake of red meat, poultry, animal offal, fish, pastries, and fried food and drinking tap water during pregnancy contributed to higher concentrations of most PFASs, after adjustment for sociodemographic characteristics and lifestyles - Higher intake of wheat, coarse cereals, tubers, and soy products was associated with lower maternal PFAS concentrations |
| --- | --- | --- | --- | --- | --- | --- | --- | --- | --- | --- | --- | --- | --- |

| Tsai et al. 2018 | Longitudinal | Japan (2003-2012) | N=2123 | -- | PFHxS  PFHxA  PFHpA  PFOS  PFOA  PFNA  PFDA  PFUnDA  PFDoDA  PFTrDA  PFTeDA | Maternal plasma | - Collected in first and third trimesters | - Age - Education - Parity - Pre-pregnancy BMI - Smoking - Alcohol consumption - Household income | Questionnaires;  Birth records | - PFAS concentrations log transformed - PFAS concentrations below MDL were assigned a value equal to half MDL - PFAS with detection rates below 50% excluded from analysis - Linear regression used to analyzed temporal trend - Used multivariable linear regression to analyze and calculate the crude and adjusted values of PFAS levels for each category - Potential confounders selected *a priori* and more than 10% change of the estimate in the model | - Maternal age at delivered - Pre-pregnancy BMI - Maternal educated - Household income - Parity | - Age at delivery positively associated with PFUnDA (p for trend=0.025, β for age ≥35=0.131 (0.036, 0.227) - Women with pre-pregnancy BMI >25 kg/m2 had lower PFDoDA ]β=-0.166 (-0.244, -0.088] and PFTrDA [β=-0.162 (-0.238, -0.087)] compared to normal BMI - Multiparity associated with lower PFHxS [β=-0.233 (-0.305, -0.16)], PFOS [β=-0.286 (-0.344, -0.227)], PFOA [-0.621 (-0.702, -0.54)], PFNA [-0.271 (-0.33, -0.212)], and PFDA [-0.135 (-0.197, -0.072)] - Education level positively associated with PFHxS (p=0.004), PFOS (p<.001), PFOA (p=0.022), PFNA (p<.001), PFDA (p<.001), PFUnDA (p=<.001), PFDoDA (p=0.005), and PFTrDA (p=0.032) - Passive smokers had higher levels of PFOS [β=0.069 (0.027, 0.111)] and PFOA [β=0.060 (0.002, 0.119)] compared to non-smokers - Alcohol consumption associated with lower levels of PFDA [β=-0.027 (-0.048, -0.006)] - Household income positively associated with PFHxS (p=0.041), PFOS (p=0.001), PFOA (p=0.003), and PFNA (p=0.004) | - Age at delivery positively associated with PFUnDA - Women with pre-pregnancy BMI >25 kg/m2 had lower PFDoDA and PFTrDA levels than did those with normal BMI - Pregnant women who were passive smokers had higher PFOS and PFOA than the non-smokers - Lower levels of PFHxS, PFOS, PFOA, PFNA, and PFDA were observed in women who had given birth to more than one child - There were significant positive associations between PFAS levels and annual income or maternal education |
| --- | --- | --- | --- | --- | --- | --- | --- | --- | --- | --- | --- | --- | --- |

| Xu et al. 2019 | Longitudinal | China (2016-2017) | N=110 dyads | Mean: 31.2; SD: 4.4 | 8:2 Cl-PFAES  6:2 Cl-PFAES  PFOS  PFHxS  PFDoA  PFUnDA  PFDA  PFNA  PFOA  PFHpA  PFHxA | Cord serum | - collected immediately following delivery | - Maternal age - Pre-pregnancy BMI - Drinking water source - Parity | Questionnaire | - Concentrations below LOQ were replaced by LOQ/sqr(2) - PFAS concentrations log-transformed - Conducted univariate and multivariable linear regressions - Potential covariates chosen *a priori* | - Maternal age - Maternal BMI - Pregnancy weight gain - Gestational age - Education - Job - Abortion times - Parity times - Birth gender - Drinking water | - Mothers with higher age at delivery (>31 years) were associated with higher levels of 6:2 Cl-PFESA (median (IQR); 0.839 (0.654) ng/mL), PFHxS (0.769 (1.07) ng/mL) and PFDoA (0.041(0.025) ng/mL) than the lower age group (≤31 years) (p <  0.05) - Primiparity higher levels of 6:2 Cl-PFESA, PFOS, PFUnDA and PFOA than multiparous women (p <  0.05) - the cord PFOS and PFUnDA were increased in mothers who directly drank tap water than tap filtered water for the p <  0.01 - Overweight participants (pre-pregnancy BMI >22.5 kg m^2^) tended to accumulate elevated 6:2 Cl-PFESA and PFOS than the regular (18.5-22.5 kg m^2^) and underweight groups (≤18.5 kg m^2^) (p <  0.05) | - Older ages, parity, source of drinking water, and higher pre-pregnancy BMI were associated with higher cord PFAS concentrations |
| --- | --- | --- | --- | --- | --- | --- | --- | --- | --- | --- | --- | --- | --- |

| Yamada et al. 2014 | Cross-sectional | France (2006, 2011, and 2010-2013) | N=106 | -- | PFOA  PFNA  PFDA  PFUnA | Maternal serum | - At birth among women with a planned caesarean section | - Marine fish and seafood consumption - Freshwater fish consumption - Dairy product consumption - Bread, crackers consumption | Questionnaire | - Normality of distributions was tested with Kolmogorov-Smirnov test and homoscedasticity with Bartlett test - Wilcoxon-Mann-Whitney test was used to compare the mean exposure between 2 groups, Kruskal-Wallis test if more than 2 groups - Ward’s method was applied for the hierarchical cluster analysis to define 3 groups of freshwater fishes | Unadjusted | - Dietary exposures were higher for pregnant women compared to the general population (p<.0001 for the 15 PFAAs) | - For all considered populations, the major exposure contributors to PFAS concentrations are fish, seafood, and water under the lower bound hypothesis, while dairy products, bread, and crispbread are the main contributors under upper bound hypothesis |
| --- | --- | --- | --- | --- | --- | --- | --- | --- | --- | --- | --- | --- | --- |

| Yang et al. 2019 | Cross-sectional | China (2013-2014) | N=534 | -- | PFOA  PFOS  PFHxS  PFUdA  PFNA | Maternal serum | - Collected in “early term of pregnancy” | - Diet pattern (cereals, beans, vegetables, fruit, meat, fish, milk, etc) - Daily intakes of food items - Total energy of individual - Age - BMI - Gestational age - Education - Career - Income - Gravidity - Parity - Smoking status | Questionnaire | - One-way ANOVA used to analyze the associations of sociodemographic factors with serum PFAAs concentrations - PFNA and PFUdA concentrations log-transformed; PFOA, PFOS, and PFHxS box-cox transformed - Multiple linear regression used to examine associations - Covariates included in model if p<.02 using a backward stepwise approach - Diet pattern classification performed by factor analysis - Each person given a component score for each dietary pattern and divided into tertiles - Three models were built for each diet pattern and PFAA | - Adjusting mentioned, but states only “energy and sociodemographic factors” | - Age associated with PFOA [β=0.008 (-0.003, 0.019)], PFOS [β=0.027 (0.006, 0.047)], PFUdA [β=0.018 (0.001, 0.034)], and PFNA [0.038 (0.018, 0.059)] - BMI associated with PFOA [β=0.010 (0.000, 0.021)], PFOS [β=0.020 (0.000, 0.039)], and PFHxS [β=0.009 (0.002, 0.016)] - Smoking associated with PFOS [β=-0.594 (-1.148, -0.039)] - Cereal consumption associated with PFHxS [β=-0.111 (-0.221, -0.001)] - Bean consumption associated with PFOS [β1.729 (0.368, 3.091)] and PFHxS [β=0.724 (0.242, 1.207)] - Aquatic product consumption associated with PFOA [β=0.394 (0.029, 0.759)] | - Age was positively associate with most PFAS: PFOA, PFOS, PFUdA, and PFNA - BMI was positively associated with PFOA, PFOS, and PFHxS - Smoking negatively associated with PFOS - Cereal consumption negatively associated with PFHxS - Bean consumption positively associated with PFHxS - Aquatic product consumption positively associated with PFOA |
| --- | --- | --- | --- | --- | --- | --- | --- | --- | --- | --- | --- | --- | --- |

*Abbreviations: BMI: body mass index; Et-PFOSA-AcOH: 2-(N-Ethyl-perfluorooctane sulfonamido) acetic acid; FOSA: perfluorooctane sulfonaminde; GFR: glomerular filtration rate; GM: geometric mean; Hg: mercury; LOD: limit of detection; Me-PFOSA-AcOH: 2-(N-Methyl-perfluorooctane sulfonamido) acetic acid; N-Et-FOSA: N-ethyl perfluorooctane sulfonamide; N-Me-FOSA: N-methylperfluoro-1-octanesulfonamide; OC: oral contraceptive; PFBA: perfluorobutanoic acid; PFBS: perfluorobtane sulfonic acid; PFDA: perfluorodecanoic acid; PFDeA: perfluorodecanoic acid; PFDoA: perfluorododecanoic acid; PFDS: perfluorodecane sulfonate; PFHpA: perfluoroheptanoic acid; PFHxA: perfluorohexanoic acid; PFHxS: perfluorohexane sulfonate; PFNA: perfluorononanoic acid; PFOA: perfluorooctanoic acid; PFOS: perfluorooctane sulfonic acid; PFOSA: perfluorooctane sulfonamide; PFPeA: perfluoropentanoic acid; PFTeDA: perfluorotetraddecanoic acid; PFSA: perfluoroalkylated sulfonate; PFTrDA: perfluorotridecanoic acid; PFUA: perfluoroundecanoic acid; PFUdA: perfluoroundecanoic acid; PFUnDA: perfluoroundecanoic acid; Se: selenium; SS: statistically significant; 6:2 Cl-PFAES: 6:2 Chlorinated polyfluorinated ether sulfonate; 8:2 Cl:PFAES: 8:2 Chlorinated polyfluorinated ether sulfonate

**PubMed Searches completed 4/1/2022-12/12/2022**

(maternal OR maternal factors OR maternal determinants OR maternal risk OR pregnant women OR determinants pregnant women) AND

(perfluoroalkyl OR polyfluoroalkyl OR

PFAS or PFOA or PFOS or PFHxS)
